# 4-Phenylbutyric acid enhances the mineralization of osteogenesis imperfecta iPSC-derived osteoblasts

**DOI:** 10.1074/jbc.RA120.014709

**Published:** 2020-11-23

**Authors:** Shinji Takeyari, Takuo Kubota, Yasuhisa Ohata, Makoto Fujiwara, Taichi Kitaoka, Yuki Taga, Kazunori Mizuno, Keiichi Ozono

**Affiliations:** 1 Department of Pediatrics, Osaka University Graduate School of Medicine, Suita, Osaka, Japan; 2 Nippi Research Institute of Biomatrix, Toride, Ibaraki, Japan

**Keywords:** endoplasmic reticulum (ER), extracellular matrix, fibril, glycosylation, osteoblast, BMD, bone mineral density, CHP, collagen hybridizing peptide, CRTAP, cartilage-associated protein, CyPB, cyclophilin B, ECM, extracellular matrix, ER, endoplasmic reticulum, GGHL, glucosyl-galactosyl-hydroxylysine, HSP47, heat shock protein 47, iPSCs, induced pluripotent stem cells, MSCs, mesenchymal stromal cells, 4-PBA, 4-phenylbutyric acid, PDI, protein disulfide isomerase, P3H1, prolyl-3-hydroxylase 1, PTMs, posttranslational modifications, OI, osteogenesis imperfecta, SDS-PAGE, sodium dodecyl sulfate–polyacrylamide gel electrophoresis, TGF-β, transforming growth factor β

## Abstract

Osteogenesis imperfecta (OI) is a heritable brittle bone disease mainly caused by mutations in the two type I collagen genes. Collagen synthesis is a complex process including trimer formation, glycosylation, secretion, extracellular matrix (ECM) formation, and mineralization. Using OI patient-derived fibroblasts and induced pluripotent stem cells (iPSCs), we investigated the effect of 4-phenylbutyric acid (4-PBA) on collagen synthesis to test its potential as a new treatment for OI. Endoplasmic reticulum (ER) retention of type I collagen was observed by immunofluorescence staining in OI patient-derived fibroblasts with glycine substitution and exon skipping mutations. Liquid chromatography–mass spectrometry analysis revealed excessive glycosylation of secreted type I collagen at the specific sites in OI cells. The misfolding of the type I collagen triple helix in the ECM was demonstrated by the incorporation of heat-dissociated collagen hybridizing peptide in OI cells. Type I collagen was produced excessively by OI fibroblasts with a glycine mutation, but this excessive production was normalized when OI fibroblasts were cultured on control fibroblast-derived ECM. We also found that mineralization was impaired in osteoblasts differentiated from OI iPSCs. In summary, treatment with 4-PBA normalizes the excessive production of type I collagen, reduces ER retention, partially improves misfolding of the type I collagen helix in ECM, and improves osteoblast mineralization. Thus, 4-PBA may improve not only ER retention, but also type I collagen synthesis and mineralization in human cells from OI patients.

Osteogenesis imperfecta (OI, OMIM # 166200, 166210, 259420, 166220) is a heritable brittle bone disease resulting from low bone mineral density and poor bone quality (1). OI is classically divided into types 1–4 based on the phenotype reported by Sillence *et al*. (2): type I is mild, type II lethal, type III severe, and type IV moderate. OI is mainly caused by autosomal dominant mutations of *COL1A1* or *COL1A2*, encoding the α1 or α2 chain of type I collagen, respectively. Variants that reduce the synthesis of type I collagen such as nonsense mutations usually result in mild OI type I, whereas variants that alter type I collagen structure such as glycine substitution mutations usually result in severe OI type III or moderate OI type IV (3, 4, 5). On the other hand, variants that cause exon skipping such as splice site mutations are associated with either mild OI type I, or severe OI type III, or moderate OI type IV (4, 6). The exon skipping in *COL1A1* or *COL1A2* is mainly in-frame, leading to the deletion of several to a dozen of amino acids. Thus, type I collagen coded by the gene with the exon skipping likely has the altered structure of the trimer. It is well known that chains with a glycine substitution in the helix region also show altered structure. These structurally altered α(I)-chains take time to assemble into a trimer in endoplasmic reticulum (ER), resulting in excessive posttranslational modifications (PTMs) (7). To some extent, overmodified collagen is secreted and incorporated into collagen fibrils, and such overmodified fibrils may contribute to defective extracellular matrix (ECM) function (8). Defective ECM changes the quantity of bone mineral density as well as other bone properties involved in bone quality, eventually leading to the severe type of OI (9, 10). As an alternative explanation for the pathogenesis, some structurally altered collagen products cause ER stress due to their retention in the ER, inducing osteoblasts with abnormal function (8).

Bisphosphonate therapy has been widely used to treat OI. This treatment reduces the bone resorption and increases bone mineral density (BMD) in many OI patients. Systematic reviews of randomized trials indicate that the prevention of fractures is limited, especially in severe cases, although bisphosphonate therapy increases BMD and decreases fracture rates to some extent (11, 12, 13, 14). This is because bone quality may not be improved enough by bisphosphonate therapy. Therefore, treatments that improve bone quality are desirable. New drugs, transforming growth factor β (TGF-β) or sclerostin neutralizing antibodies, are now under development for OI (15, 16, 17). Although there are less data compared with these antibodies, 4-phenylbutyric acid (4-PBA) is also being considered as a new drug for treating OI. 4-PBA, an ammonia scavenger, is FDA-approved for treating urea cycle disorders (18). Regarding OI, amelioration of bone mineralization and skeletal deformities of zebrafish OI models by 4-PBA has been reported in 2017 (19). Furthermore, 4-PBA suppressed ER enlargement and improved cellular homeostasis of human dermal fibroblasts from collagenous and noncollagenous OI patients (20, 21). We independently searched for a suitable drug for treating OI and discovered 4-PBA. We applied for a patent in 2016 and received the patent for 4-PBA related to the normalizing of excessive mutant type I collagen in ER, from the Japan patent office in 2018 (patent number is JP6429401). Although many studies have reported on the chemical chaperone function of 4-PBA in relation to several disorders, it remains unclear whether 4-PBA exerts other effects in addition to that of a chemical chaperone (22).

Collagen synthesis is a complex process including trimer formation of two α1(I)-chains and one α2(I)-chain, PTMs, secretion, ECM formation, and mineralization. The current study examined these complex type I collagen syntheses in a step-by-step manner using OI patient-derived dermal fibroblasts, as well as osteoblasts differentiated from OI patient-specific induced pluripotent stem cells (iPSCs). We also examined the effect of 4-PBA on these processes.

## Results

### ER retention of type I collagen in OI fibroblasts

**Figure 1:**
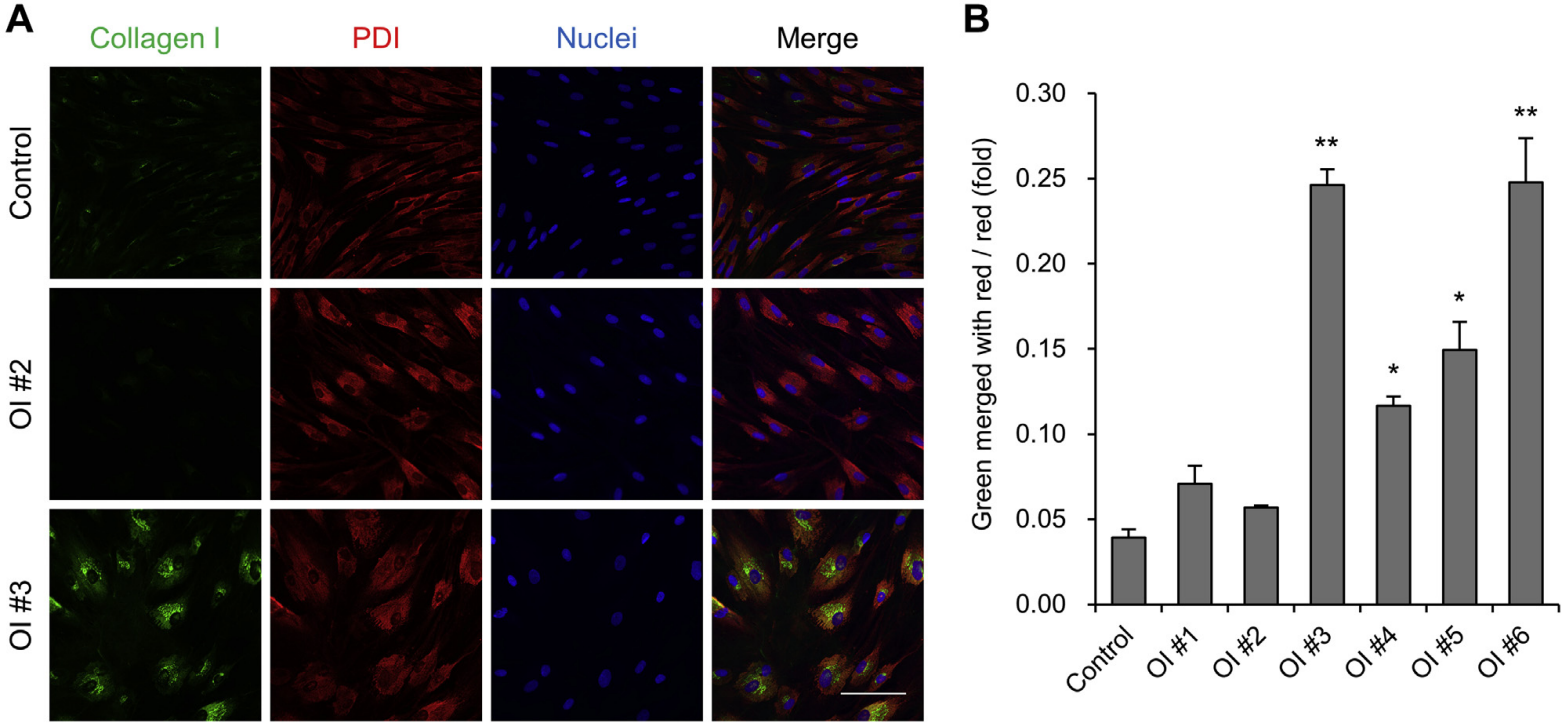
**Intracellular localization of type I collagen.***A*, immunofluorescence staining of dermal fibroblasts. Type I collagen is in *green*. Endoplasmic reticulum marker protein, disulfide isomerase (PDI), is in *red*. Nuclei are in *blue*. Scale bar is 100 μm. *B*, calculated values of *green area* merged with *red area* divided by *red area* of immunofluorescence staining. Data are mean ± SEM. Differences were tested by ANOVA followed by Tukey's HSD post hoc test. ∗*p* < 0.05 *versus* control, ∗∗*p* < 0.01 *versus* control.

We examined colocalization of type I collagen and protein disulfide isomerase (PDI), an ER marker, of control and six OI patient-derived dermal fibroblasts by immunofluorescence staining (Fig. 1*A* and Fig. S1). Disease variants and characteristics of OI patients are listed in Table 1. Control and mild type OI #1 and 2 had almost no colocalization of type I collagen and PDI, whereas in severe type OI #3–6, type 1 collagen and PDI significantly colocalized (Fig. 1, *A*–*B* and Fig. S1). However, the expression of *BIP* and *CHOP*, which encode unfolded protein response markers, did not increase (Fig. S2), suggesting no so-called ER stress. ER retention of type I collagen was confirmed in OI fibroblasts with a glycine substitution mutation and a severe-type exon skipping mutation. Conversely, OI fibroblasts with a nonsense mutation and a mild-type exon skipping mutation did not show ER retention of type I collagen.

**Table 1:** Characteristics of patients

Patient number	1	2	3	4	5	6
Sillence classification	Type I	Type I	Type III	Type III	Type III	Type III
Gene	*COL1A1*	*COL1A1*	*COL1A1*	*COL1A1*	*COL1A2*	*COL1A2*
Variant	c.2667+1G>C	c.495T>G	c.2461G>A	c.1615-2A>T	c.3305G>T	c.1090–3_1155del
Variant effect	Exon 38 skipping	p.Tyr165∗	p.Gly821Ser	Exon 24 skipping	p.Gly1102Val	Exon 21 skipping
Blue sclera	+	+	+	+	+	+
Dentinogenesis imperfecta	−	−	+	+	+	+
Age at first visit	4y5m	4y5m	0y3m	0y1m	3y0m	0y0m
Height at first visit (cm)	99.2	98.4	51.0	44.9	83.7	39.5
Height SDS	−0.92	−0.98	−4.10	−3.67	−2.53	−4.28
BMD of L1 to L4 (g/cm^2^)	0.765	0.880	0.313	0.344	0.835	0.620
BMD Z score	−1.70	−0.75	−5.99	−4.61	−0.61	0.10

BMD, bone mineral density; *COL1A1*: RefSeq NG_007400.1, NM_000088.3; *COL1A2*: RefSeq NG_007405.1, NM_000089.3.

### Overmodification of type I collagen in OI fibroblasts

**Figure 2:**
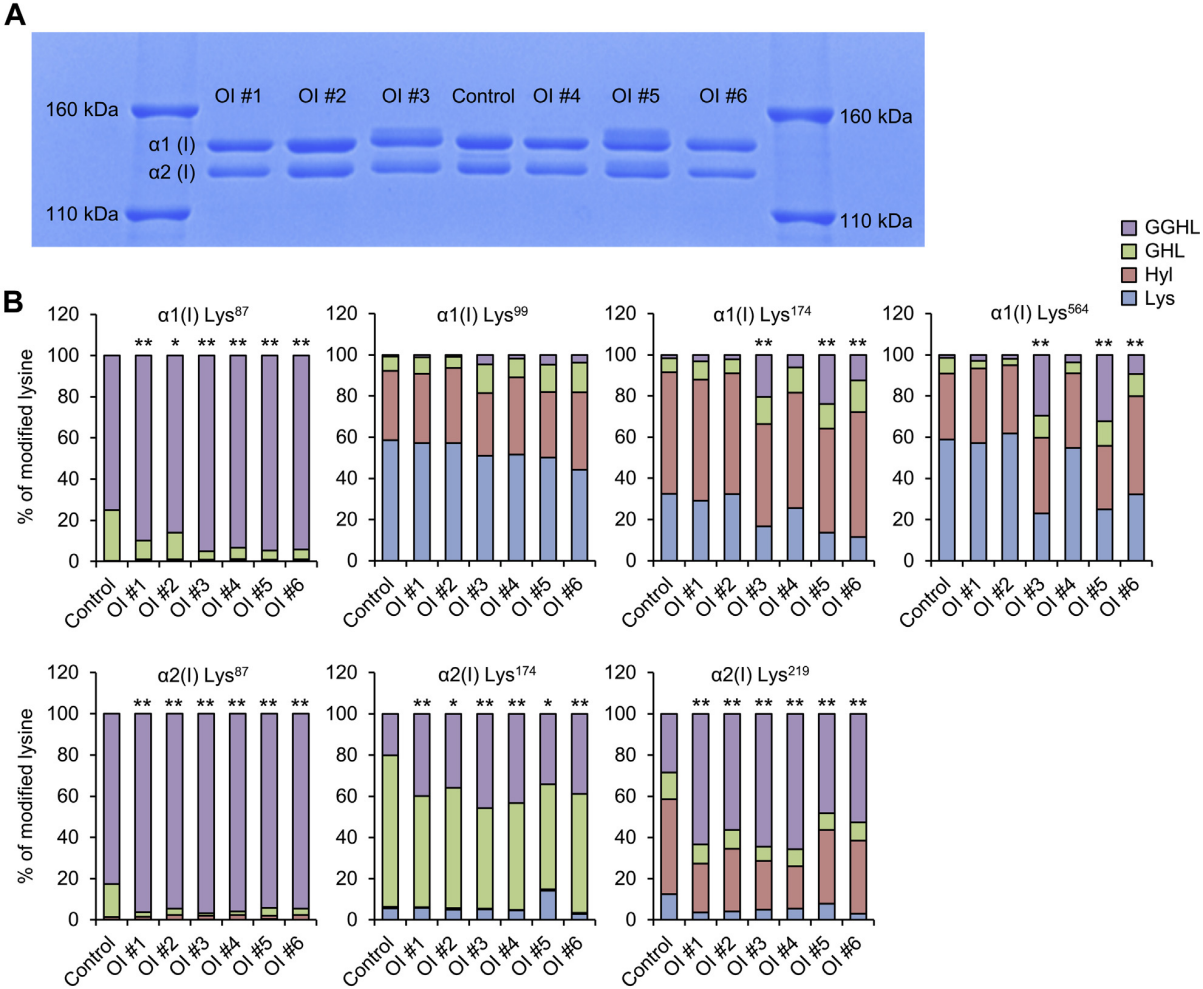
**Posttranslational modification of type I collagen.***A*, SDS-PAGE analysis of type I collagen in conditioned medium. *B*, percentage of posttranslational modifications at Lys^87^, Lys^99^, Lys^174^, and Lys^564^ of α1 and Lys^87^, Lys^174^, and Lys^219^ of the α2 chain of type I collagen analyzed by liquid chromatography–mass spectrometry. Control is the average of 2 cell lines. Differences in the ratio of GGHL to non-GGHL were tested by Pearson's Chi-square test. ∗*p* < 0.05 *versus* control, ∗∗*p* < 0.01 *versus* control. GGHL, glucosyl-galactosyl-hydroxylysine; GHL, galactosyl-hydroxylysine; Hyl, hydroxylysine.

To assess PTMs, mobility of α1(I) and α2(I) bands was evaluated using sodium dodecyl sulfate–polyacrylamide gel electrophoresis (SDS-PAGE); (Fig. 2*A*). OI #1, 2, 4, and 6 with a nonsense or an exon skipping mutation displayed the same mobilities as the control, but OI #3 and 5 with a glycine substitution mutation displayed retarded bands of α1(I) and α2(I), suggesting that some α1(I) and α2(I) chains are overglycosylated. Next, we analyzed PTMs of seven positions in the helical domain, including Lys^87^, Lys^99^, Lys^174^, and Lys^564^ of α1(I) and Lys^87^, Lys^174^, and Lys^219^ of α2(I) via liquid chromatography–mass spectrometry (LC-MS) (Fig. 2*B*). Although SDS-PAGE did not show significant overglycosylation in OI #1, 2, 4, and 6, the percentage of glucosyl-galactosyl-hydroxylysine (GGHL) at Lys^87^ of α1(I) and Lys^87^, Lys^174^, and Lys^219^ of α2(I) was higher in all OI cells than in control ones. Lys^87^ is known to be important for the formation of covalent cross-linking between the nonhelical region of one molecule and the helical region of another (23). In addition, at Lys^174^ and Lys^564^ of α1(I), the percentage of GGHL was much higher in OI #3 and 5. It is likely that the overglycosylation of these positions contributed to the retarded bands observed during SDS-PAGE. Taken together, in addition to overglycosylation of Lys^174^ and Lys^564^ in OI cells with a glycine substitution mutation, Lys^87^ of α1(I) and α2(I) was overglycosylated in all OI cells.

### Excess production of type I collagen in OI fibroblasts with a glycine substitution mutation owing to impaired ECM

**Figure 3:**
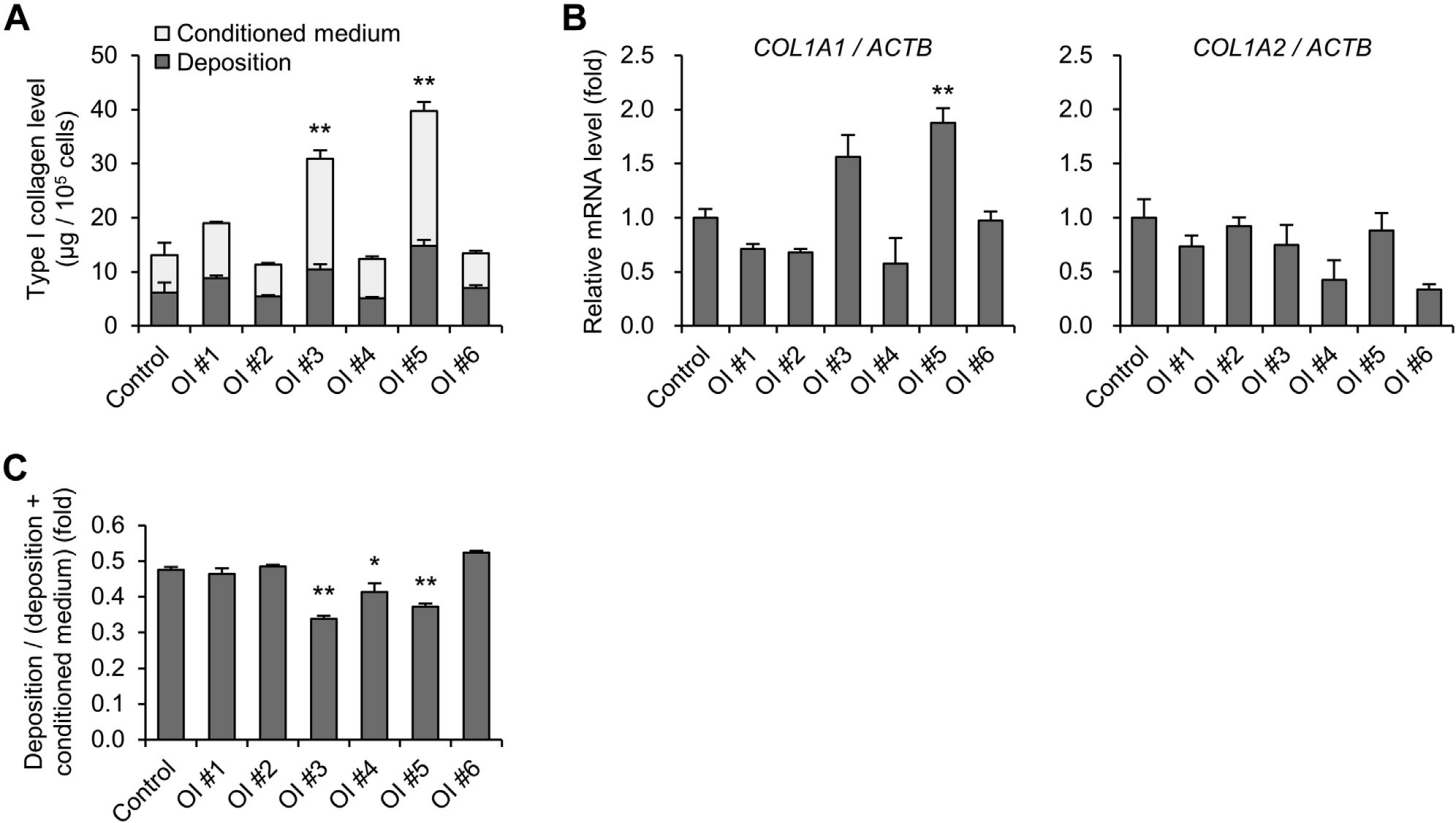
**Production of type I collagen.***A*, protein levels of type I collagen in conditioned medium and deposited on the dish measured by ELISA. *B*, real-time quantitative PCR of *COL1A1* and *COL1A2*. *C*, calculated values of protein levels of type I collagen. The formula for calculation is as follows: protein levels deposited on the dish/(protein levels deposited on the dish + protein levels in conditioned medium). All data are mean ± SEM. Differences were tested by ANOVA followed by Tukey's HSD post hoc test. ∗*p* < 0.05 *versus* control, ∗∗*p* < 0.01 *versus* control.

Enzyme-linked immunosorbent assay (ELISA) was used to evaluate the amount of type I collagen protein in the conditioned medium and ECM deposited on the culture dish (Fig. 3*A*). Following the secretion of type 1 collagen trimers into the medium by cells, type I collagen was deposited on the dish as collagen fibrils as components of the ECM. Type I collagen secreted from fibroblasts of OI #3 and 5 was significantly higher than that from the control. The mRNA expression level of *COL1A1* from OI #5 fibroblasts was increased compared with that from the control, and that from OI #3 fibroblasts showed a tendency to be increased (Fig. 3*B*). The expression levels of *COL1A2* showed no obvious difference between OI and control fibroblasts. Although there was a difference in *COL1A1/COL1A2* expression ratio among OI fibroblasts (Fig. S3*A*), there was no difference in the percentage of α1(I) and α2(I) chains in conditioned medium quantified by LC-MS (Fig. S3*B*). On the other hand, the amount of type I collagen protein in the ECM deposited on the culture dish in OI #3 and 5 was not elevated compared with that in the control, even though the amount of secreted type I collagen in OI #3 and 5 was significantly higher than that in the control. We also calculated the ratio of type I collagen protein levels deposited on the dish to that in conditioned medium and deposited on the dish (Fig. 3*C*). The ratio of deposited type I collagen was actually low in OI #3 and 5. It was also low in OI #4.

**Figure 4:**
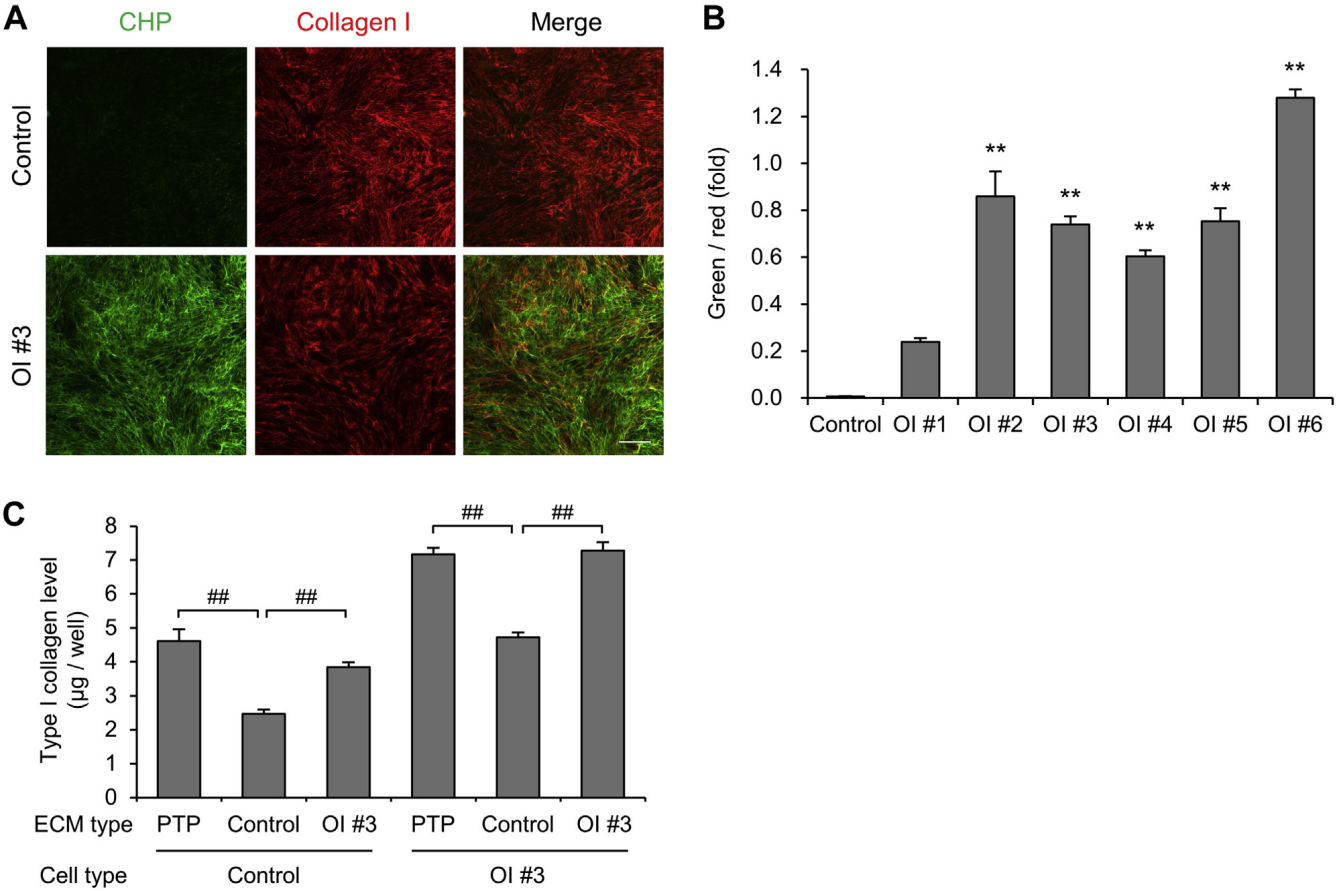
**Detection of impaired type I collagen in extracellular matrix (ECM).***A*, fluorescence staining of deposited collagen. Misfolded triple-helical chains are stained in *green* by collagen hybridizing peptide (CHP). Type I collagen is stained in *red*. Scale bar is 200 μm. *B*, calculated values of fluorescence staining. The formula for calculation is as follows: (*green area* × green fluorescence density)/(*red area* × red fluorescence density). *C*, protein levels of type I collagen in conditioned medium. Control and OI #3 fibroblasts were cultured on plasma-treated polystyrene plates, control fibroblast-derived ECM, or OI #3 fibroblast-derived ECM. After a 24-h incubation period, the type I collagen level in conditioned medium was measured. All data are mean ± SEM. Differences were tested by ANOVA followed by Tukey's HSD post hoc test. ∗∗*p* < 0.01 *versus* control, ##*p* < 0.01.

We found that type I collagen secreted from OI fibroblasts was not efficiently deposited, suggesting some sort of structural defectiveness of type I collagen. To detect the structurally defective triple helix of type I collagen in the ECM, we used a collagen hybridizing peptide (CHP) and compared the area stained by CHP and type I collagen antibodies (Fig. 4, *A*–*B*, Fig. S4). CHP is a commercially available peptide that may specifically bind to misfolded collagen chains such as heat-denatured type 1 collagen. The ECM derived from control fibroblasts was not stained by CHP at all, whereas the ECM derived from OI fibroblasts was significantly stained by CHP, except for that derived from OI #1. The ECM derived from OI #1 fibroblasts also tended to be stained by CHP compared with that from control fibroblasts. These results indicate that the ECM derived from OI fibroblasts contained misfolded type I collagen chains.

To further detect the abnormal ECM and examine its role, we seeded control or OI #3 fibroblasts on control or OI #3 fibroblast-derived ECM or plasma-treated polystyrene plates and measured type I collagen levels in the conditioned medium (Fig. 4*C*). Interestingly, type I collagen levels of both control and OI #3 fibroblasts were decreased on the control fibroblast-derived ECM, but not on the OI #3 fibroblast-derived ECM, compared with those on plasma-treated polystyrene plates. These results suggested that control ECM functions as a signal to produce type I collagen in a negative feedback manner and that impaired ECM produced by OI #3 fibroblasts fails to reduce the production of type I collagen. In a sense, the defective ECM is involved in excessive production and/or less ECM assembly of secreted type I collagen.

### The reduction of ER retention but almost no change in overmodification of type I collagen by 4-PBA

**Figure 5:**
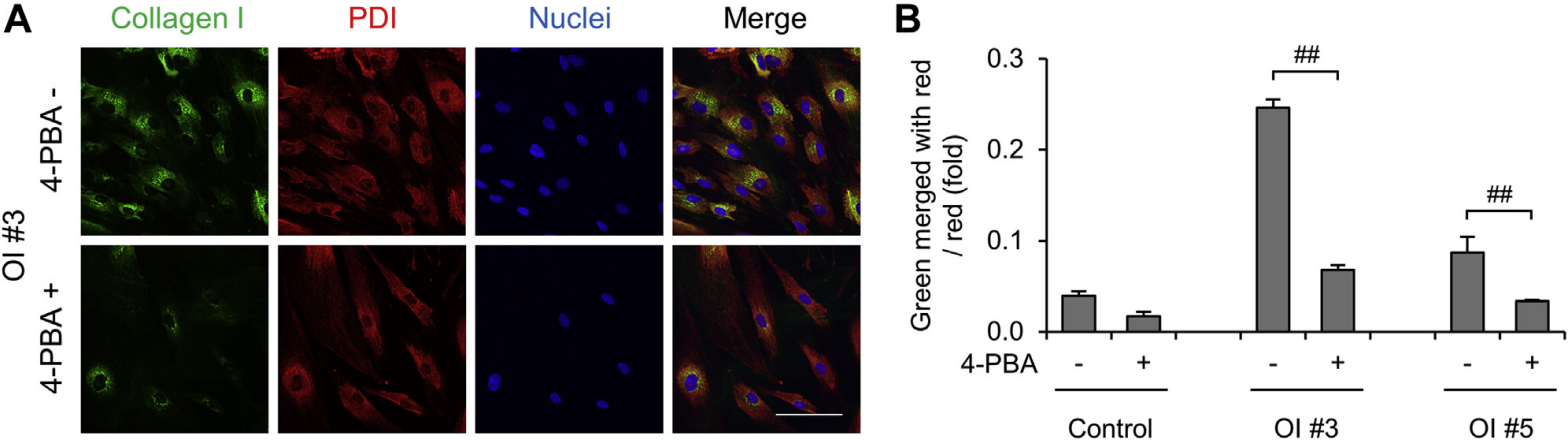
**The effect of 4-phenylbutyric acid (4-PBA) on intracellular accumulation of type I collagen.** Each cell line was treated with or without 5 mM 4-PBA. *A*, immunofluorescence staining of dermal fibroblasts. Type I collagen is in *green*. The endoplasmic reticulum marker protein, disulfide isomerase (PDI), is in *red*. Nuclei are in blue. Scale bar is 100 μm. *B*, calculated values of *green area* merged with *red area* divided by red area of immunofluorescence staining. Data are mean ± SEM. Differences were tested by ANOVA followed by Tukey's HSD post hoc test. ##*p* < 0.01.

ER retention and overmodification of type I collagen were observed in OI fibroblasts, especially in OI with a glycine mutation. Next, we treated control and OI fibroblasts with 4-PBA and examined its effects on these events especially in fibroblasts of OI #3 and 5. ER retention of type I collagen was significantly reduced by treatment with 4-PBA in fibroblasts of OI #3 and 5 (Fig. 5, *A*–*B*). For the other OI fibroblasts, ER retention of type I collagen was significantly reduced by treatment with 4-PBA except for OI #2 (Fig. S5, *A*–*B*). However, 4-PBA showed slight effects on overmodification of type I collagen (Fig. S6). Improvement of overglycosylation was found only in Lys^87^ of α2(I) of OI #5. Additionally, the retarded bands of α1(I) and α2(I) of OI #3 were not apparently changed by 4-PBA in SDS-PAGE (data not shown). These results showed that treatment with 4-PBA reduced ER retention but did not much improve overglycosylation of type I collagen in OI fibroblasts.

### The improvement of excessive production of type I collagen and the decrease in the ratio of misfolded type I collagen helix in ECM by 4-PBA

**Figure 6:**
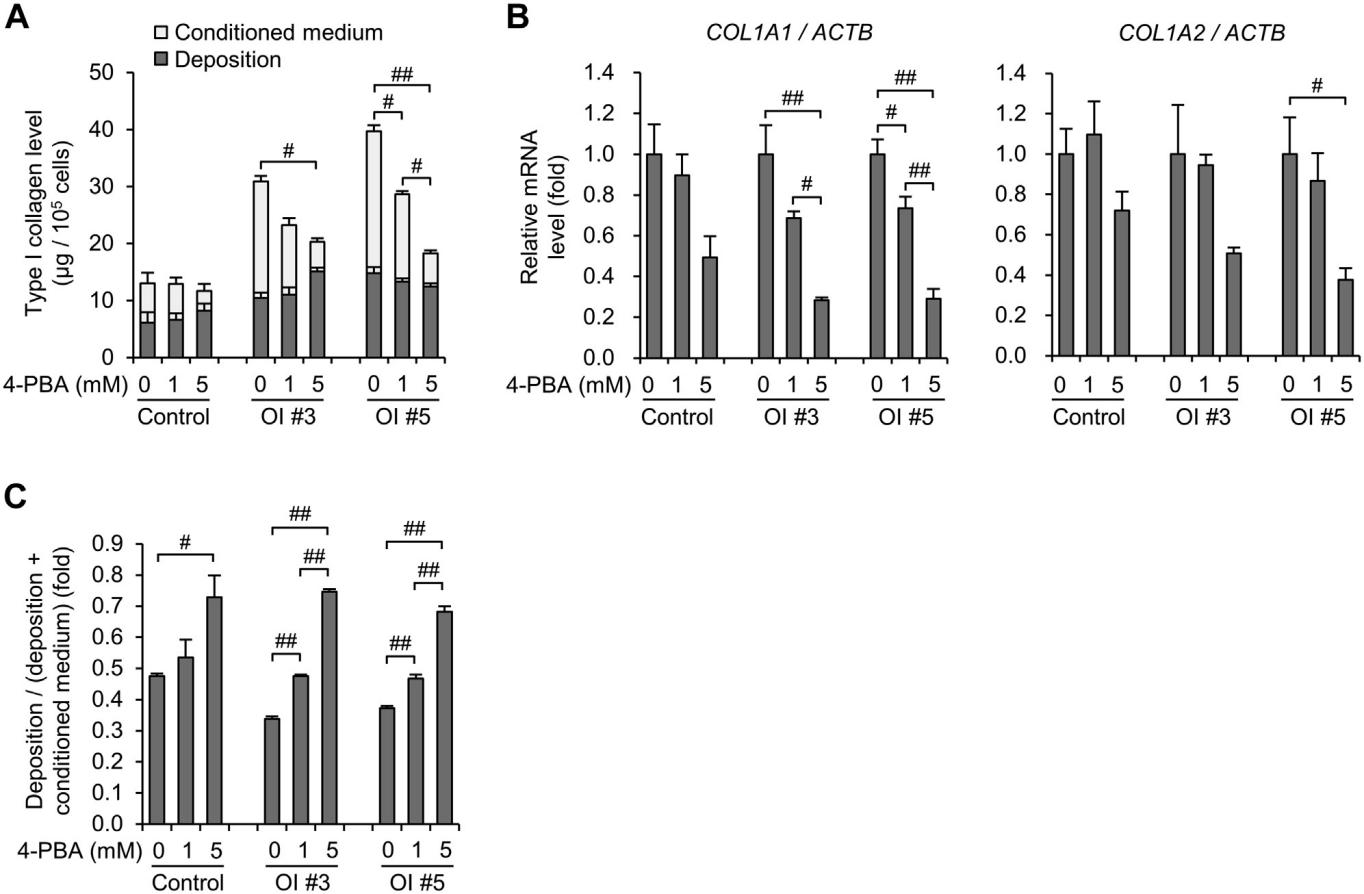
**The effect of 4-phenylbutyric acid (4-PBA) on the production of type I collagen.***A*, protein levels of type I collagen in conditioned medium and deposited on the dish measured by ELISA. *B*, real-time quantitative PCR of *COL1A1* and *COL1A2*. *C*, calculated values of protein levels of type I collagen. The formula for calculation is as follows: protein levels deposited on the dish/(protein levels deposited on the dish + protein levels in conditioned medium). All data are mean ± SEM. Differences were tested by ANOVA followed by Tukey's HSD post hoc test. #*p* < 0.05, ##*p* < 0.01.

**Figure 7:**
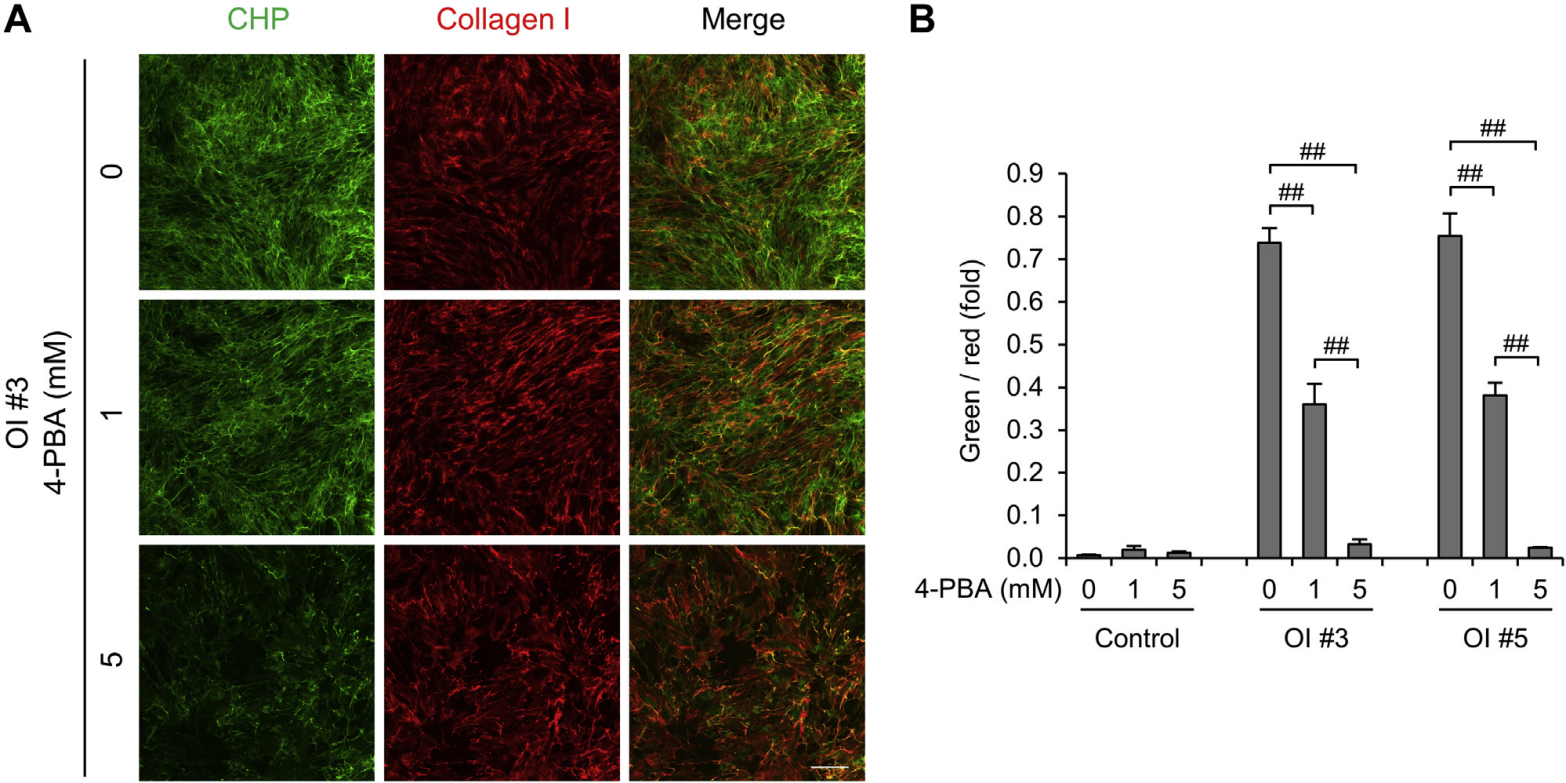
**The effect of 4-phenylbutyric acid (4-PBA) on type I collagen in the extracellular matrix.***A*, fluorescence staining of deposited collagen. Misfolded triple-helical chains are stained in *green* by collagen hybridizing peptide (CHP). Type I collagen is stained red. Scale bar is 200 μm. *B*, calculated values of fluorescence staining. The formula for calculation is as follows: (*green area* × green fluorescence density)/(*red area* × red fluorescence density). Data are mean ± SEM. Differences were tested by ANOVA followed by Tukey's HSD post hoc test. ##*p* < 0.01.

We examined the protein level of type I collagen secreted by fibroblasts treated with 4-PBA. The type I collagen level secreted by the fibroblasts of OI #3 and 5 was significantly decreased by 4-PBA to control levels, whereas that from control fibroblasts was not altered (Fig. 6*A*). Although 4-PBA also altered the type I collagen levels of other OI fibroblasts, the change was small compared with that of OI #3 and 5 (Fig. S7A). Consistent with the protein levels of type I collagen, gene expression of *COL1A1* of OI #3 and 5 and *COL1A2* of OI #5 was also significantly decreased, and *COL1A2* gene expression of OI #3 had a tendency to be decreased (Fig. 6*B*). We calculated the ratio of type I collagen protein levels deposited on the dish to that in conditioned medium and deposited on the dish (Fig. 6*C*). The ratio was significantly increased in OI #3 and 5 by 4-PBA more efficiently compared with that in the control. It was also increased in other OI (Fig. 7*B*).

Because type I collagen secreted from OI fibroblasts was efficiently deposited on the culture dish by 4-PBA, we investigated its structure in ECM derived from fibroblasts treated with 4-PBA. 4-PBA decreased the normal type I collagen chains to some extent, but it drastically decreased the misfolded type I collagen chains stained by CHP in OI #3 and 5 (Fig. S8). Consequently, 4-PBA significantly decreased the ratio of the misfolded type I collagen chains to the normal in OI #3 and 5 (Fig. 7, *A*–*B*). These data suggested that 4-PBA treatment improved the quality of the structure of type I collagen chains in ECM. The ratio of misfolded type I collagen chains to correctly folded chains in ECM was also decreased by 4-PBA in other OI (Fig. S9, *A*–*B*).

### Impaired mineralization of induced osteoblasts in OI with a glycine substitution mutation and the improvement of the mineralization by 4-PBA

**Figure 8:**
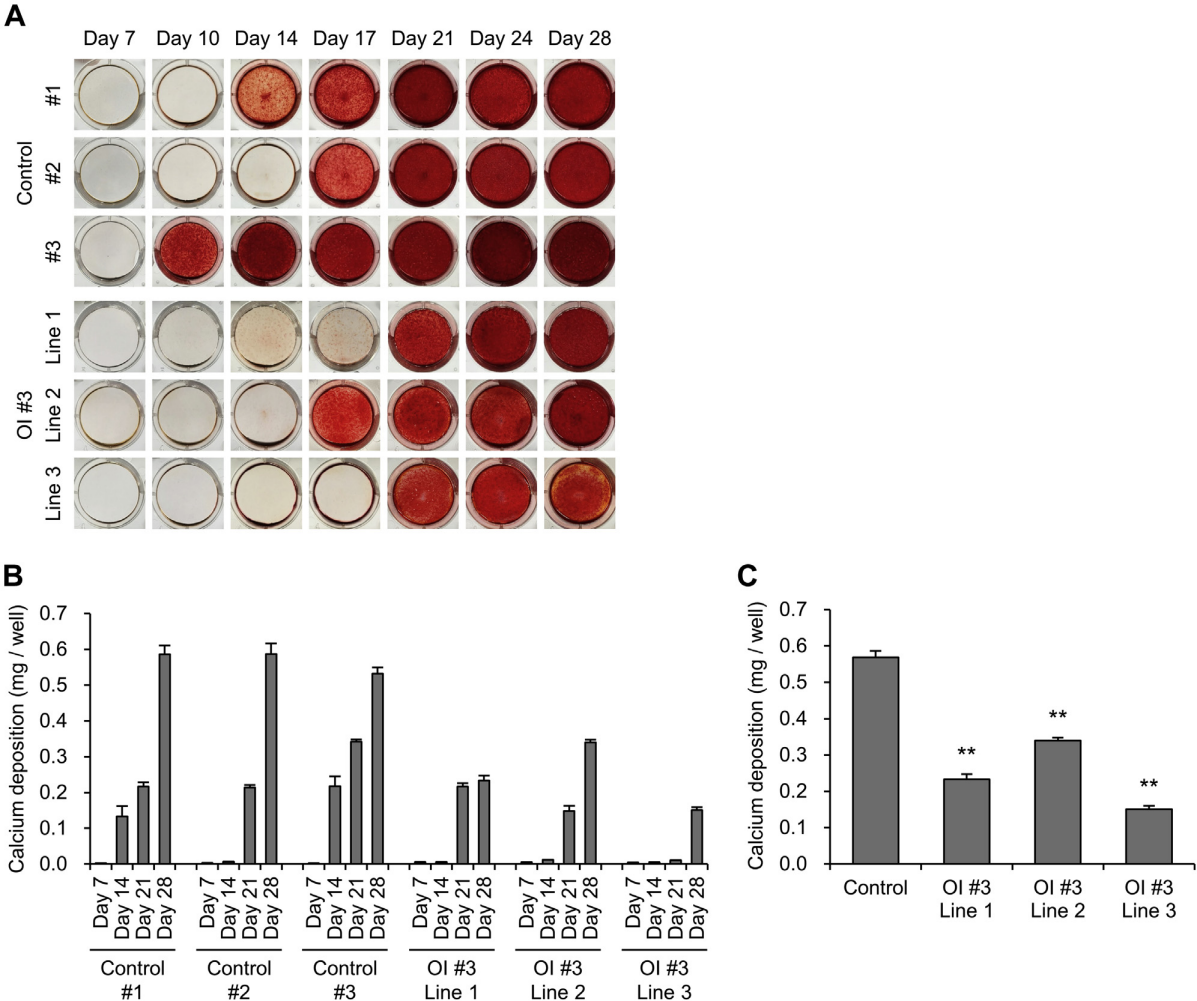
**Assessment of calcium deposition of osteoblasts differentiated from induced pluripotent stem cells.***A*, alizarin red S staining of deposited extracellular matrix derived from induced osteoblasts. Osteoblast differentiation from mesenchymal stromal cells was performed during the indicated day. *B*, the amount of calcium deposition of induced osteoblasts. Calcium deposition was quantified by a calcium assay kit at 7, 14, 21, and 28 days of osteoblast differentiation culture. *C*, calcium deposition amount, measured by a calcium assay kit, of induced osteoblasts at 28 days of osteoblast differentiation culture. All data are mean ± SEM. Differences were tested by ANOVA followed by Tukey's HSD post hoc test. ∗∗*p* < 0.01 *versus* control.

**Figure 9:**
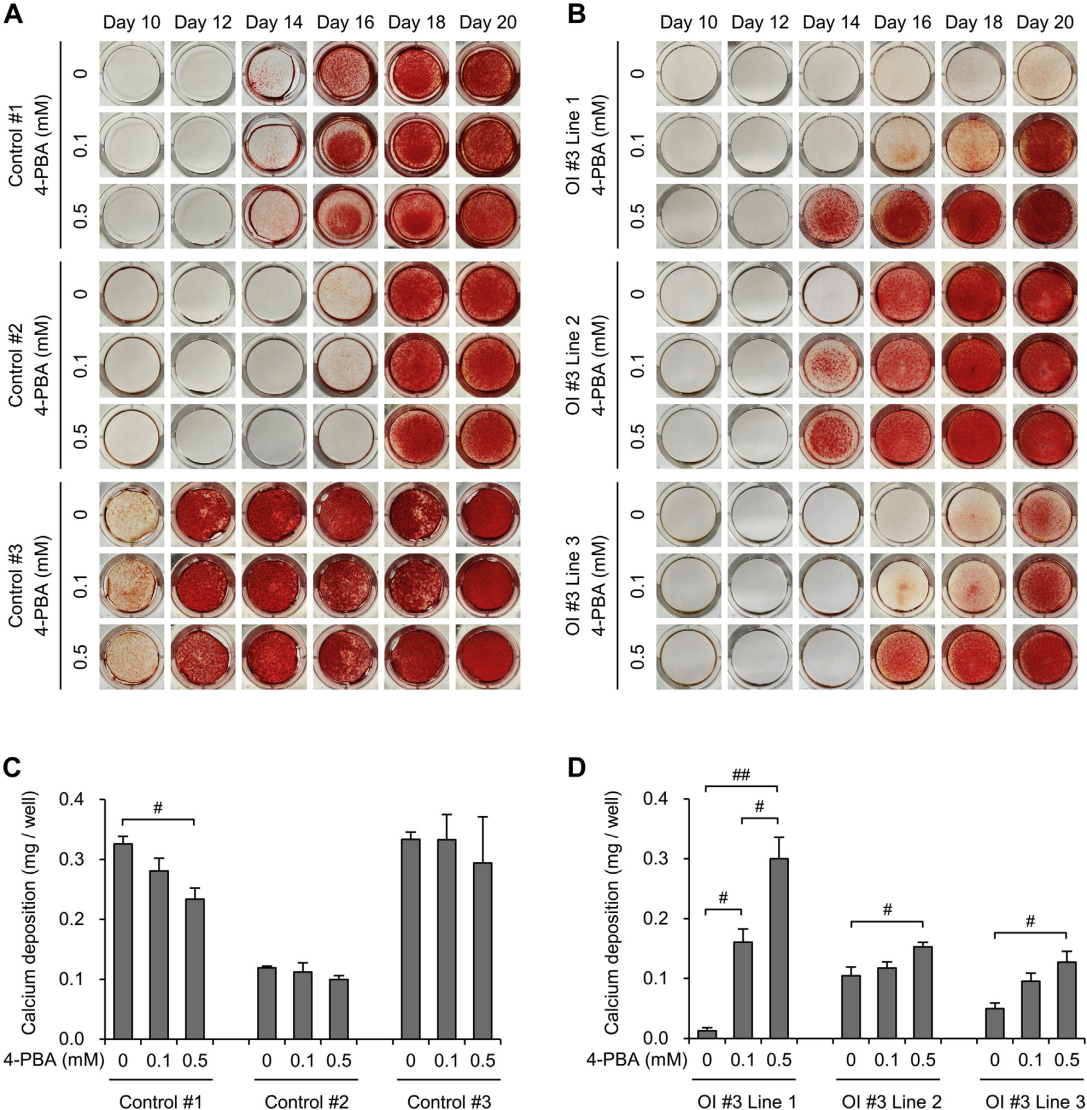
**The effect of 4-phenylbutyric acid (4-PBA) on calcium deposition of osteoblasts differentiated from induced pluripotent stem cells.***A* and *B*, alizarin red S staining of deposited extracellular matrix derived from control (*A*) and OI #3 (*B*)-induced osteoblasts. Osteoblast differentiation culture from mesenchymal stromal cells was performed during the indicated day. *C* and *D*, calcium deposition amount, measured by a calcium assay kit, of induced osteoblasts of control (*C*) and OI #3 (*D*) at 16 days of osteoblast differentiation culture. Data are mean ± SEM. Differences were tested by ANOVA followed by Tukey's HSD post hoc test. #*p* < 0.05, ##*p* < 0.01.

We investigated the impaired ECM derived from OI fibroblasts and the effect of 4-PBA on it. To investigate the ECM derived from osteoblasts, we generated iPSCs from OI #3 fibroblasts and induced differentiation of these into osteoblasts. The differentiation of iPSCs into osteoblasts was confirmed via osteogenic gene expression levels (Fig. S10). Among the osteogenic genes, *ALPL*, which is involved in the formation of hydroxyapatite, seemed to have lower expression levels in OI #3-induced osteoblasts than in the control. We then examined the expression of pro- and anti-calcification genes (*ALPL*, *PARP1*, *PARP2* (24, 25), *versus NT5E*, *ENPP1*, *ANKH*) and protein levels of alkaline phosphatase in control and OI #3-induced osteoblasts. Both the gene and protein expression levels of alkaline phosphatase in OI #3-induced osteoblasts were lower than those in the control, and there were significant differences in all other genes except for *PARP2* between the control and OI #3-induced osteoblasts (Fig. S11*A* and Fig. S12, *A*–*B*). Next, we analyzed mineralization of the ECM derived from control and OI #3-induced osteoblasts, via alizarin red S staining and quantification of calcium content (Fig. 8, *A*–*B*). Mineralization was detected 10–17 days following the induction of osteoblast differentiation in control iPSCs, and the amount of calcium, quantified using a calcium assay kit, was increased during the culture. On the other hand, mineralization was detected 17–21 days following osteoblast differentiation of OI #3 iPSCs. OI #3-induced osteoblasts needed a longer time period to begin mineralization. Furthermore, the amount of calcium deposition in OI #3 was significantly smaller than that in the control at day 28 following osteoblast differentiation (Fig. 8*C*). These data indicated that osteoblasts induced from OI #3 showed impaired mineralization. Furthermore, we investigated the effect of 4-PBA on mineralization and found that 4-PBA remarkably promoted mineralization in all lines of OI #3 (Fig. 9, *B*–*D*). On the other hand, 4-PBA did not promote mineralization in osteoblasts induced from control iPSCs (Fig. 9, *A*–*C*). To confirm that 4-PBA enhanced mineralization by itself, or via the effect on type I collagen production and hence improvement of mineral nucleation sites in the ECM, we performed alkaline phosphatase staining (Fig. S13) and investigated the expression of several genes involved in mineralization as well as the protein levels of alkaline phosphatase in the control and OI #3-induced osteoblasts with or without 4-PBA (Fig. S11*B* and Fig. S12, *A* and *C*). Alkaline phosphatase was well stained at day 14 following osteoblast differentiation, and there was no apparent difference between OI #3-induced osteoblasts with and without 4-PBA.

## Discussion

In the present study, we demonstrated abnormalities of type I collagen synthesis in OI fibroblasts and impaired mineralization of OI osteoblasts induced from patient-specific iPSCs. Administration of 4-PBA to these cells improved the abnormalities in culture. The beneficial effects of 4-PBA on ER retention and the phenotypes of zebrafish OI models have been reported (19, 20, 21). Besio *et al*. (21) focused on the effect of 4-PBA on cellular homeostasis, whereas we focused on type I collagen synthesis. Thus, the current study provides new data pertaining to the effects of 4-PBA on type I collagen synthesis, in addition to its chaperone function in OI fibroblasts and induced osteoblasts.

ER retention of type I collagen was observed in a mouse OI model, Aga2, with a dominant frameshift mutation in the collagen type I α1 C-propeptide domain (26) and a dachshund OI model with a mutation in the heat shock protein 47 (HSP47) gene, which encodes a chaperone for collagen biosynthesis in the ER (27). ER enlargement was observed in α2(I)-G610C mouse OI models (28) and zebrafish OI models (19) as well as in mouse Aga2 models (26), consistent with the observation in human dermal fibroblasts with a glycine substitution mutation in type I collagen (21) and a variant in prolyl-3-hydroxylase 1 (P3H1), cartilage-associated protein (CRTAP), and cyclophilin B (CyPB) (20). There is no doubt that certain types of OI, which carry structurally defective type I collagen or defectively folded type I collagen, show ER retention of type I collagen. For OI with an exon skipping mutation, there are no studies reporting ER retention of type I collagen to the best of our knowledge, even though splice site mutations, which may lead to exon skipping, account for 20% of *COL1A1* or *COL1A2* helical mutations identified in OI patients (3). Besides, OI with an exon skipping mutation represents a broad range of severity, and the genotype–phenotype correlation remains unclear. Remarkably, we demonstrated ER retention of type I collagen in severe-type OI with an exon skipping mutation, but mild-type OI with an exon skipping mutation did not show significant ER retention of type I collagen. These data suggested an association between ER retention and severity of OI to some extent.

Nascent type I procollagen undergoes several PTMs during the formation of the triple-helical structure in the ER. Hence, type I procollagen with structural or folding defects is exposed to PTM enzymes for a longer time, resulting in overmodification (8, 29). PTMs, especially prolyl 4-hydroxylation, are important for proper folding, transport, and ECM assembly, but the precise functions of these are yet to be revealed (8, 30). Although the precise mechanism by which overmodification of type I collagen has an impact on the phenotype of OI patients remains unclarified, one explanation reported previously was the disturbance of the growth of hydroxyapatite crystals by overglycosylation, resulting in bone brittleness (9, 10). Overmodification of type I collagen in severe type OI has been demonstrated via electrophoresis for over 30 years (31). Electrophoresis enables investigation of the molecular weight of type I collagen under varying degrees of glycosylation. Because LC-MS allows to investigate PTMs of lysine at various positions (32), we assessed total and respective modification of lysine via SDS-PAGE and LC-MS, respectively. As a result, LC-MS could detect overmodification of type I collagen more sensitively than SDS-PAGE. In OI with a nonsense mutation, type I collagen is supposed to show no structural defects, but the overall production amount is reduced. Because reduced production leads to an increase in PTM enzymes relative to collagen synthesis, overmodification of type I collagen may occur (33). For the OI with an exon skipping mutation, our data are the first to indicate overmodification of type I collagen. In type I collagen synthesis, two α1 chains and one α2 chain assemble into a trimer from C-propeptides. Hence, α chain translated from mRNA with an exon skipping mutation may become short. If one or two short α chains are incorporated into a trimer, a proper structure may not be built in the triple helix region near N-propeptides. Therefore, the trimer may be exposed to PTM enzymes for a longer period, resulting in overmodification.

Our data showed excessive production of type I collagen in OI fibroblasts with a glycine substitution mutation. Although the disease variants responsible for developing OI were different, osteocytes of *oim* and *Crtap* mice also demonstrated overexpression of *Col1a1* and *Col1a2* (34). Since a feedback regulation of type I collagen via α2β1 integrin has been reported (35, 36), impaired ECM formation may cause the excessive production of type I collagen. Because some of the type I collagen produced excessively may have had structural defectiveness due to a glycine substitution mutation, there was difficulty in their assembly and deposition on the culture dish (Fig. 3*C*), and ECM containing mutant type I collagen might be associated with the excessive production of type I collagen (Fig. 4*C*), resulting in a vicious circle.

We detected the impaired collagen triple helix in ECM using CHP, which binds to misfolded collagen chains such as denatured type I collagen. Although we are unable to reveal the precise mechanism, the results that CHP bound to type I collagen in OI with an exon skipping mutation suggest that the triple helix of type I collagen secreted by fibroblasts of OI with an exon skipping mutation is structurally abnormal. In OI with an exon skipping mutation, a short α chain is likely translated because of an in-frame deletion. When the slightly short α chain is incorporated into a trimer, triple helix formation is not tight enough, allowing CHP to bind to the helix. It is more difficult to explain why CHP bound to type I collagen secreted by fibroblasts for OI with a nonsense mutation than for OI with an exon skipping mutation. It is considered that the pathogenesis of OI with a nonsense mutation is reduced amount of type I collagen synthesis but not structural abnormality of type I collagen. One possibility is that in OI #2 cells with c.495T>G mutation (p.Tyr165∗) in *COL1A1*, typical nonsense-mediated decay might not be induced as most other nonsense mutations because the expression of *COL1A1* and the amount of secreted type I collagen were not reduced in OI #2 fibroblasts (Fig. 3, *A*–*B*). Therefore, the pathogenesis of OI #2 might be different from that of most other nonsense mutations, and OI #2 may have some sort of structural abnormality in type I collagen.

We demonstrated the multiple effects exerted by 4-PBA on type I collagen synthesis improvement in OI fibroblasts. First, 4-PBA decreased the excessive production of type I collagen in OI fibroblasts with a glycine substitution mutation. It is still unclear as to how 4-PBA reduces the amount of secreted type I collagen. One conceivable mechanism is that 4-PBA directly downregulates the expression of *COL1A1* and *COL1A2*, while another suggested mechanism is that the chaperon activity of 4-PBA facilitates the ER degradation of misfolded collagen. Either way, 4-PBA reduces type I collagen synthesis, resulting in the reduction of type I collagen in the ER. This is consistent with previous reports that 4-PBA restores ER enlargement in OI cells (19, 20, 21). It is speculated that reduction of ER retention of type I collagen results in improving ER function, which, in turn, exerts beneficial effects on type I collagen quality. Despite the reduction of ER retention, there was almost no effect of 4-PBA on overmodification of type I collagen. ER retention, protein folding, and overglycosylation of type I collagen may be associated in OI cells (8, 37), but it is unclear which of these processes occurs first.

In addition to the chaperone effect, 4-PBA may also alter the quality of type I collagen. Our data showed remarkable improvement in the ratio of misfolded type I collagen helixes using CHP in OI fibroblasts. Improvement in ER function may lead to a decrease in the secretion of misfolded type I collagen rather than correcting the morphologically defective type I collagen. Furthermore, the deposition rate of secreted type I collagen was also increased by 4-PBA (Fig. 6*C*), indicating that 4-PBA may enhance type I collagen to easily assemble into fibrils and deposit on the dish. Altogether, 4-PBA may improve the quality of type I collagen.

Finally, mineralization is the last important step in the functioning of osteoblasts. Impaired mineralization in OI osteoblasts differentiated from iPSCs has been previously described, although the protocol pertaining to osteoblast differentiation was different (38). It is difficult to precisely declare whether impaired mineralization in OI-induced osteoblasts was due to deterioration of mineralization activity or impairment of osteoblast differentiation. However, following a 28-day incubation period, which allowed sufficient time for differentiation into osteoblasts, the amount of calcium deposition in all OI #3 lines was smaller than that in the control. A previous study showed intact expression of osteogenic genes and impaired mineralization in OI-induced osteoblasts (38). Thus, impaired mineralization in OI #3 with a glycine substitution mutation might be caused by the deterioration of mineralization activity and not from the impairment of osteoblast differentiation. In contrast to our data of remarkable improvement in mineralization in OI #3-induced osteoblasts, 4-PBA was reported to have the effect of preventing calcification of vascular smooth muscle cells (39) and valvular interstitial cells (40) by inhibiting ER stress. The difference in cell types between our study and previous study may explain the calcification-promoting effect of 4-PBA in induced osteoblasts in our data. Considering the effect of 4-PBA on fibroblast-derived ECM, it appears that 4-PBA may have improved the quality of type I collagen produced from OI #3-induced osteoblasts and changed the ECM, thus facilitating mineralization.

In summary, we demonstrated ER retention and overmodification of type I collagen and misfolded type I collagen helixes in OI fibroblasts. Excessive production of type I collagen in OI with a glycine substitution mutation was observed, which might be due to impaired ECM. A new finding is that OI with a glycine substitution mutation may have not only qualitative but also quantitative defect in type I collagen. In osteoblast differentiated from OI iPSCs with a glycine substitution mutation, mineralization was impaired. We also demonstrated several effects of 4-PBA on OI cells. 4-PBA enhanced the impaired mineralization of induced OI osteoblasts with a glycine substitution mutation. In addition, 4-PBA substantially decreased ER retention of type I collagen and misfolded type I collagen helixes in OI fibroblasts. These beneficial effects of 4-PBA were considered to be the result of decreased type I collagen production. Although the detailed mechanism of how 4-PBA affects type I collagen synthesis remains to be elucidated and *in vivo* data of OI model animals are needed, it is likely that 4-PBA may be a promising drug that exerts effects other than those exerted by bisphosphonate. In conclusion, we analyzed the multistep type I collagen synthesis in OI fibroblasts and induced osteoblasts and demonstrated the beneficial effects of 4-PBA.

## Experimental procedures

### Ethical approval

Ethical approval, including iPSCs production, was obtained from Osaka University Graduate School of Medicine Ethical Committee (approval number: 10196 (775-2)-3), and all the studies were performed according to the Declaration of Helsinki. Informed consent was obtained from the parents or legal guardians of the patients.

### Subjects

Six Japanese patients with OI were included in this study. Each patient carried different heterozygous mutations in either *COL1A1* (n = 4) or *COL1A2* (n = 2) gene. Mutations and characteristics of the patients are listed in Table 1. BMD data were collected following bisphosphonate treatment, except during the first visit. The Z score for BMD was calculated according to reference values relative to age, sex, and race (41). Each variant was confirmed via direct sequencing using genomic DNA from blood samples. OI #1 with the c.2667+1G>C may lead to exon 38 skipping, resulting in an 18-amino-acid deletion. OI #4 with the c.1615-2A>T may lead to exon 24 skipping, resulting in an 18-amino-acid deletion. Precise exon 21 skipping of OI #6 with the c.1090-3_1155del mutation was confirmed by direct sequencing using total RNA from cultured dermal fibroblasts, and exon 21 skipping may lead to a 36-amino-acid deletion.

### Isolation and culture of human dermal fibroblasts

We obtained dermal fibroblasts from OI patients at the time of surgery. Control human dermal fibroblasts isolated from neonatal foreskin (Gibco, Grand Island, NY) were used as normal controls. All fibroblasts were maintained in Dulbecco Modified Eagle's Medium (D-MEM) high glucose with L-glutamine, 10% fetal bovine serum (FBS), 25 U/ml penicillin, and 25 μg/ml streptomycin at 37 °C in a humidified 5% CO_2_ incubator. During each experiment, control and OI fibroblasts were plated at a described density and cultured in D-MEM high glucose with L-glutamine, 2% FBS, 200 μM L-ascorbic acid phosphate magnesium salt n-hydrate (Wako, Tokyo, Japan), 25 U/ml penicillin, and 25 μg/ml streptomycin, for collagen synthesis.

### Immunofluorescence staining of intracellular type I collagen

OI and control fibroblasts were plated on a 96-well plate at a density of 4.0 × 10^3^ cells/well in triplicate and cultured in collagen synthesis medium. Following a 4-day incubation period, with or without 5 mM 4-PBA (Sigma, St Louis, MO), cells were washed with PBS thrice, fixed with 4% paraformaldehyde for 10 min at room temperature followed by ice-cold 100% methanol for 1 min at −20 °C, washed with PBS thrice, and incubated in blocking buffer (1% BSA, 10% FBS, 0.3 M glycine, 0.1% Tween 20 in PBS) for 1 h at room temperature. Next, cells were incubated with type I collagen antibodies (Abcam, Cambridge, UK #ab34710) diluted 1:200 and PDI antibodies (Enzo, New York, NY #ADI-SPA-891) diluted 1:200 in blocking buffer for 1.5 h at room temperature. After being washed with PBS thrice, cells were incubated with secondary antibodies (AlexaFluor 488 conjugated anti-rabbit IgG, Jackson ImmunoResearch, West Grove, PA and Cy3 conjugated antimouse IgG, Jackson ImmunoResearch, West Grove, PA) diluted 1:500 and Hoechst 33342 solution (Dojindo, Kumamoto, Japan) diluted 1:100 in blocking buffer for 1 h at room temperature. After being washed with PBS thrice, fluorescence images from 25 fields/well were scanned using an automated IN Cell Analyzer 6000 microscope (GE Healthcare, Bucks, UK). For quantification of type I collagen accumulated in ER, we calculated the ratio of type I collagen area merged with PDI area to PDI area, using IN Cell Investigator software (GE Healthcare, Bucks, UK).

### SDS-PAGE analysis of type I collagen in conditioned medium

OI and control fibroblasts were plated on a 10-cm dish at a density of 1.0 × 10^6^ cells/dish. After being cultured in maintenance medium until full confluence was reached, cells were cultured in collagen synthesis medium for 3 days with or without 5 mM 4-PBA. Collagen was purified from the culture medium via pepsin digestion (0.1 mg/ml in 0.1 N HCl; Sigma, St Louis, MO) and salt precipitation (1 M NaCl) as previously described (42). Purified collagen was denatured at 95 °C for 3 min, electrophoresed on 8% polyacrylamide gel, and stained using Coomassie Brilliant Blue R-250 (Bio-Rad, Hercules, CA). The stained gel was destained in water with 40% methanol and 10% acetic acid, and an image of stained collagen was obtained using ChemiDoc XRS Plus (Bio-Rad, Hercules, CA).

### Site-specific characterization of lysine PTMs in type I collagen

OI and control fibroblasts were plated on a 10-cm dish at a density of 1.0 × 10^6^ cells/dish. After being cultured in maintenance medium until full confluence was reached, cells were cultured in collagen synthesis medium for 3 days with or without 5 mM 4-PBA. Collagen was purified from the culture medium via pepsin digestion (0.1 mg/ml in 0.1 N HCl) and salt precipitation (1 M NaCl) as previously described (42). Following heat denaturation at 60 °C for 30 min, the purified collagen samples were digested with sequencing grade-modified trypsin (Promega, Madison, WI; 1:50 enzyme/substrate ratio) in 100 mM Tris-HCl and 1 mM CaCl_2_ (pH 7.6) at 37 °C for 16 h. The tryptic digests were subjected to LC-MS analysis on a maXis II quadrupole time-of-flight mass spectrometer (Bruker Daltonics, Billerica, MA) coupled to a Shimadzu Prominence UFLC-XR system (Shimadzu, Kyoto, Japan) with an Ascentis Express C18 HPLC column (5 μm particle size, L × I.D. 150 mm × 2.1 mm; Supelco, Bellefonte, PA) (43). MS scan was performed in positive ion mode with data acquisition using otofControl version 4.0 (Bruker Daltonics, Billerica, MA). Site occupancy of each lysine modification site in type I collagen was calculated using the peak area ratio of monoisotopic extracted ion chromatograms of peptides containing the respective molecular species, as previously reported for mouse type I collagen (43) with adaptation for human sequences. To determine peaks of peptides containing lysine glycosylation sites whose sequence is different from that of mouse type I collagen, tandem mass spectrometry (MS/MS) analysis was also performed for several control and OI collagen samples. The acquired MS/MS data were searched against the UniProtKB/Swiss-Prot database (release 2018_05) for *Homo sapiens* species (20,349 protein entries) using ProteinPilot software 4.0 (AB Sciex, Foster City, CA). Search parameters included digestion by trypsin, biological modification ID focus, and 95% protein confidence threshold. Search criteria for PTMs were optimized for collagen analysis as described previously (32). We manually confirmed the sequence and modification for glycosylated peptides that were not identified by the database search (Fig. S14).

### Estimation of alpha chain ratio of type I collagen

Chain ratio of type I collagen was estimated by LC-MS as described previously (44). In brief, stable isotope-labeled collagen (SI-collagen) (45) was mixed with the culture medium of control and OI fibroblasts as an internal standard, and pepsin digestion and salt precipitation were performed to purify collagen. The collagen samples were digested with trypsin (Promega, Madison, WI) in 100 mM Tris-HCl/1 mM CaCl_2_ (pH 7.6) at 37 °C for 16 h after heat denaturation at 60 °C for 30 min. The tryptic digests were analyzed by LC-MS in multiple reaction monitoring mode using a 3200 QTRAP hybrid triple quadrupole/linear ion trap mass spectrometer (AB Sciex, Foster City, CA) coupled to an Agilent 1200 Series HPLC system (Agilent Technologies, Palo Alto, CA) with a BIOshell A160 Peptide C18 HPLC column (5 μm particle size, L × I.D. 150 mm × 2.1 mm; Supelco, Bellefonte, PA). The molar concentrations of α1(I) and α2(I) chains were determined based on the peak area ratio of previously established specific marker peptides (two peptides for each chain) (45) relative to corresponding stable isotopically heavy ones derived from SI collagen.

### Measurement of type I collagen protein level

OI and control fibroblasts were plated on a 10-cm dish at a density of 1.0 × 10^6^ cells/dish in triplicate and cultured in collagen synthesis medium with or without 1 or 5 mM 4-PBA. After a 7-day incubation period with one medium change at day 3, conditioned medium was directly used as the sample, while collagen in ECM deposited on the dish was extracted using a modified version of the manual of Human Collagen Type I ELISA kit (ACEL, Kanagawa, Japan). Briefly, the cell layer was washed with PBS, scraped, digested overnight with 0.1 mg/ml of pepsin in 0.1 M HCl at 4 °C on a rotary shaker, and centrifuged at 250*g* for 15 min, and the supernatant was used as the sample after salting out with 1 M NaCl. The type I collagen levels in the conditioned medium and the ECM were measured using a Human Collagen Type I ELISA kit according to the manual.

### Real-time quantitative PCR

OI and control fibroblasts were plated on a 6-well plate at a density of 1.6 × 10^5^ cells/well in triplicate and cultured in collagen synthesis medium. After a 7-day incubation period with or without 1 or 5 mM 4-PBA, total RNA was extracted via a RNeasy Mini Kit (Qiagen, Hilden, Germany) and cDNA was synthesized from total RNA using ReverTra Ace qPCR RT Master Mix (Toyobo, Osaka, Japan) according to the manual. Real-time quantitative PCR (qPCR) was performed with a set of specific primers (Table S1) using THUNDERBIRD SYBR qPCR Mix (Toyobo, Osaka, Japan) and QuantStudio 7 Flex Real-time PCR System (Applied Biosystems, Framingham, MA). Quantification of gene expression was based on the 2^−ΔΔCt^ method and normalized to *ACTB* gene expression.

### Fluorescence staining of type I collagen in ECM

OI and control fibroblasts were plated on a 96-well plate at a density of 6.4 × 10^3^ cells/well in triplicate and cultured in collagen synthesis medium. After a 7-day incubation period with or without 1 or 5 mM 4-PBA, cells were washed with PBS thrice, fixed with 4% paraformaldehyde for 10 min at room temperature, washed with PBS thrice, incubated in blocking buffer (3% BSA in PBS) for 1 h at room temperature, and washed with PBS thrice. Next, the cells were incubated with 20 μM heat-dissociated collagen hybridizing peptide (CHP, 3Helix, Salt Lake City, UT) and type I collagen antibodies (Abcam, Cambridge, UK) diluted 1:200 in PBS for 2 h at 4 °C. After being washed thrice with PBS, cells were incubated with secondary antibodies (DyLight 549 conjugated anti-rabbit IgG, Jackson ImmunoResearch, West Grove, PA) diluted 1:500 in blocking buffer (1% BSA in PBS) for 1 h at room temperature. After being washed thrice with PBS, fluorescence images from 4 fields/well were scanned using an automated microscope IN Cell Analyzer 6000 (GE Healthcare, Bucks, UK). To compare CHP and type I collagen antibody signals, we calculated fluorescence staining values using IN Cell Investigator software (GE Healthcare, Bucks, UK). The formula used for calculation was as follows: (green area × green fluorescence density)/(red area × red fluorescence density).

### Fibroblast-derived ECM

OI and control fibroblasts were plated on a 6-well plate at a density of 1.6 × 10^5^ cells/well in triplicate and cultured in collagen synthesis medium. Fibroblast-derived ECM was obtained according to a previously reported method (35). After a 14-day incubation period, with or without 5 mM 4-PBA from day 3, the cell layer was washed with PBS and lysed with 5 M NH_3_ in PBS for 1.5 min at room temperature. The fibroblast-derived ECM was air-dried and washed with collagen synthesis medium. New control and OI fibroblasts were seeded on the fibroblast-derived ECM at a density of 1.6 × 10^5^ cells/well and cultured in collagen synthesis medium. After a 24-h incubation period, the type I collagen level in conditioned medium was measured using a Human Collagen Type I ELISA kit (ACEL, Kanagawa, Japan).

### Generation of human iPSCs, osteoblast differentiation, and analysis of mineralization

Human iPSCs of control and OI #3 were induced from dermal fibroblasts, according to a previously reported method whereby OCT4, SOX2, KLF4, and c-MYC were introduced via a Sendai virus (SeV) vector (46, 47, 48). Osteogenic differentiation from iPSCs was performed according to a previously reported method (49). Briefly, neural crest cells were induced from iPSCs with a chemically defined medium containing 10 μM SB431542 (Selleck Chemicals, Houston, TX) and 1 μM CHIR99021 (LC Laboratories, Woburn, MA), sorted using an anti-p75 antibody (BD Biosciences, Franklin Lakes, NJ) by FACS, and differentiated into mesenchymal stromal cells (MSCs) in αMEM, 10% FBS, 5 ng/ml FGF2 (Wako, Tokyo, Japan), 25 U/ml penicillin, and 25 μg/ml streptomycin. MSCs were differentiated into osteoblasts in MEM-α GlutaMAX (Gibco, Grand Island, NY), 10% FBS, 0.1 mM dexamethasone (Wako, Tokyo, Japan), 50 mg/ml ascorbic acid (Wako, Tokyo, Japan), and 10 mM β-glycerophosphate (Sigma, St Louis, MO) on laminin (Nippi, Tokyo, Japan)-coated palates for 4 weeks with or without 0.1 or 0.5 mM 4-PBA. Expression of pluripotency markers, *OCT4* and *NANOG*, MSC markers, *CD44* and *NT5E*, and osteogenic markers, *RUNX2*, *ALPL*, and *COL1A1* was checked at each step using real-time qPCR with a set of specific primers (Table S1). The expression of pro- and anticalcification genes, *ALPL*, *PARP1*, *PARP2*, *NT5E ENPP1*, and *ANKH*, was also checked at day 14 following osteoblast differentiation. Mineralization of cell layers was assessed via alizarin red S staining. The cell layer was washed with PBS, fixed with 10% neutral buffered formalin for 10 min at room temperature, washed with water, stained with 2% alizarin red S (Sigma, St Louis, MO) for 10 min at room temperature, and washed several times with water. Calcium content of cell layers was quantified by a calcium assay kit (Metallogenics, Chiba, Japan) according to the manufacturer's instructions. Briefly, each cell layer was washed with PBS and scraped. Calcium in the cell layer was extracted with 0.1 M HCl for 1 h at room temperature on a rotary shaker and centrifuged at 10,000*g* for 15 min, and the supernatant was used as the sample after being neutralized with NaOH.

### Western blotting

Osteogenic differentiation from human iPSCs of control and OI #3 was performed as mentioned above. MSCs were differentiated into osteoblasts as mentioned above for 2 weeks with or without 0.5 mM 4-PBA. Total proteins were extracted from induced osteoblasts in RIPA buffer (Wako, Tokyo, Japan) containing protease and phosphatase inhibitor cocktail (Wako, Tokyo, Japan). After centrifugation (15,000*g*, 15 min, 4 °C), supernatants were stored at −80 °C. Protein concentration was measured using a colorimetric assay kit (Bio-Rad, Hercules, CA). Proteins were subjected to SDS-PAGE and transferred to a PVDF membrane (Bio-Rad, Hercules, CA). After blocking, the membrane was incubated with primary antibody against alkaline phosphatase (R&D Systems, Minneapolis, MN #AF2910) diluted 1:200 and HRP-conjugated anti-β-actin antibody (MBL, Nagoya, Japan #PM053–7) diluted 1:5000. After incubation with the secondary antibody, the signals were visualized using ECL system (GE Healthcare, Bucks, UK). Densitometry was quantified with ImageJ 1.50i (National Institutes of Health, Bethesda, MD).

### Alkaline phosphatase staining

Alkaline phosphatase staining was performed using Sigma Fast 5-bromo-4-chloro-3-indolyl phosphate/nitro blue tetrazolium (BCIP/NBT) tablets (Sigma, St Louis, MO). Osteogenic differentiation from human iPSCs of control and OI #3 was performed as mentioned above. MSCs were differentiated into osteoblasts as mentioned above for 2 weeks with or without 0.5 mM 4-PBA. Cells were washed with PBS, fixed with 10% neutral buffered formalin for 1 min at room temperature, washed with washing buffer (0.05% Tween 20 in PBS), stained with BCIP/NBT substrate solution prepared by dissolving one tablet in 10 ml water for 10 min in the dark at room temperature, washed with washing buffer, and then PBS was added.

### Statistics

The results are presented as the mean ± SEM or percentage. Differences were tested using ANOVA followed by Tukey's HSD post hoc test or Pearson's chi-square test, as appropriate, using JMP software version 14.3.0 (SAS Institute Inc., Cary, NC). Significance was accepted at *p*-values < 0.05.

## Data availability

The MS data sets for determination of peaks of peptides containing lysine glycosylation sites have been deposited to the ProteomeXchange consortium via the jPOST partner repository with the data set identifier PXD021411.

## Conflict of interest

The authors declare that they have no conflicts of interest with the contents of this article.
