# Supplementary material for: 4-Phenylbutyric acid enhances the mineralization of osteogenesis imperfecta iPSC-derived osteoblasts
Source: J Biol Chem. 2020 Nov 23;296:100027. doi: 10.1074/jbc.RA120.014709 (PMC7948972; doi:10.1074/jbc.RA120.014709)
Supplement: Table S1 and Figures S1 to S14 [file mmc1.pdf]

4-phenylbutyric acid enhances the mineralization of osteogenesis imperfecta iPSC-derived osteoblasts

Shinji Takeyari[1], Takuo Kubota[1]*, Yasuhisa Ohata[1], Makoto Fujiwara[1], Taichi Kitaoka[1], Yuki Taga[2], Kazunori Mizuno[2], Keiichi Ozono[1]

[1]Department of Pediatrics, Osaka University Graduate School of Medicine, Suita, Osaka 565-0871, Japan
[2] Nippi Research Institute of Biomatrix, Toride, Ibaraki 302-0017, Japan

*Corresponding author: Takuo Kubota
Email: tkubota@ped.med.osaka-u.ac.jp

Supporting information list

Table S1
Figure S1
Figure S2
Figure S3
Figure S4
Figure S5
Figure S6
Figure S7
Figure S8
Figure S9
Figure S10
Figure S11
Figure S12
Figure S13
Figure S14

Table S1. The list of primer sequences used for real-time quantitative PCR

Gene		Primer sequence
COL1A1	Forward	5'- GGGATTCCCTGGACCTAAAG -3'
	Reverse	5'- GGAACACCTCGCTCTCCA -3'
COL1A2	Forward	5'- AGAGGACCACGTGGAGAAAG -3'
	Reverse	5'- GGCCTGTGGGACCATCTT -3'
ACTB	Forward	5'- TCAAGATCATTGCTCCTCCTGAG -3'
	Reverse	5'- ACATCTGCTGGAAGGTGGACA -3'
BIP	Forward	5'- TGTTCAACCAATTATCAGCAAACTC -3'
	Reverse	5'- TTCTGCTGTATCCTCTTCACCAGT -3'
CHOP	Forward	5'- AGAACCAGGAAACGGAAACAGA -3'
	Reverse	5'- TCTCCTTCATGCGCTGCTTT -3'
OCT4	Forward	5'- TGTACTCCTCGGTCCCTTTC -3'
	Reverse	5'- TCCAGGTTTTCTTTCCCTAGC -3'
NANOG	Forward	5'- CAGTCTGGACACTGGCTGAA -3'
	Reverse	5'- CTCGCTGATTAGGCTCCAAC -3'
CD44	Forward	5'- ATGGACAAGTTTTGGTGGCAC -3'
	Reverse	5'- AATACACCTGCAAAGCGGCA -3'
NT5E	Forward	5'- CACTATCTGGTTCACCGTGTACA -3'
	Reverse	5'- CGATCAGTCCTTCCACACCAT -3'
RUNX2	Forward	5'- TAGCAAGGTTCAACGATCTGAG -3'
	Reverse	5'- AGCTTCTGTCTGTGCCTTCTG -3'
ALPL	Forward	5'- CCTCGTTGACACCTGGAAGAG -3'
	Reverse	5'- TTCCGTGCGGTTCCAGA -3'
PARP1	Forward	5'- AGACAGTGTGCAGGCCAAG -3'
	Reverse	5'- ACTTCCTGATGATCTCGGCTTC -3'
PARP2	Forward	5'- GATGGTATGCCAGGAAGGTCA -3'
	Reverse	5'- TGTTGAACTGGAGATTGGTCTGA -3'
ENPP1	Forward	5'- CACTACAGCATTGTCACCGGAT -3'
	Reverse	5'- GGCCTTGATACTTAGCTGTGACC -3'
ANKH	Forward	5'- CTCTGTCACTCACGCTCTGT -3'
	Reverse	5'- TCTTCAGTGTCATCAGCCACC -3'

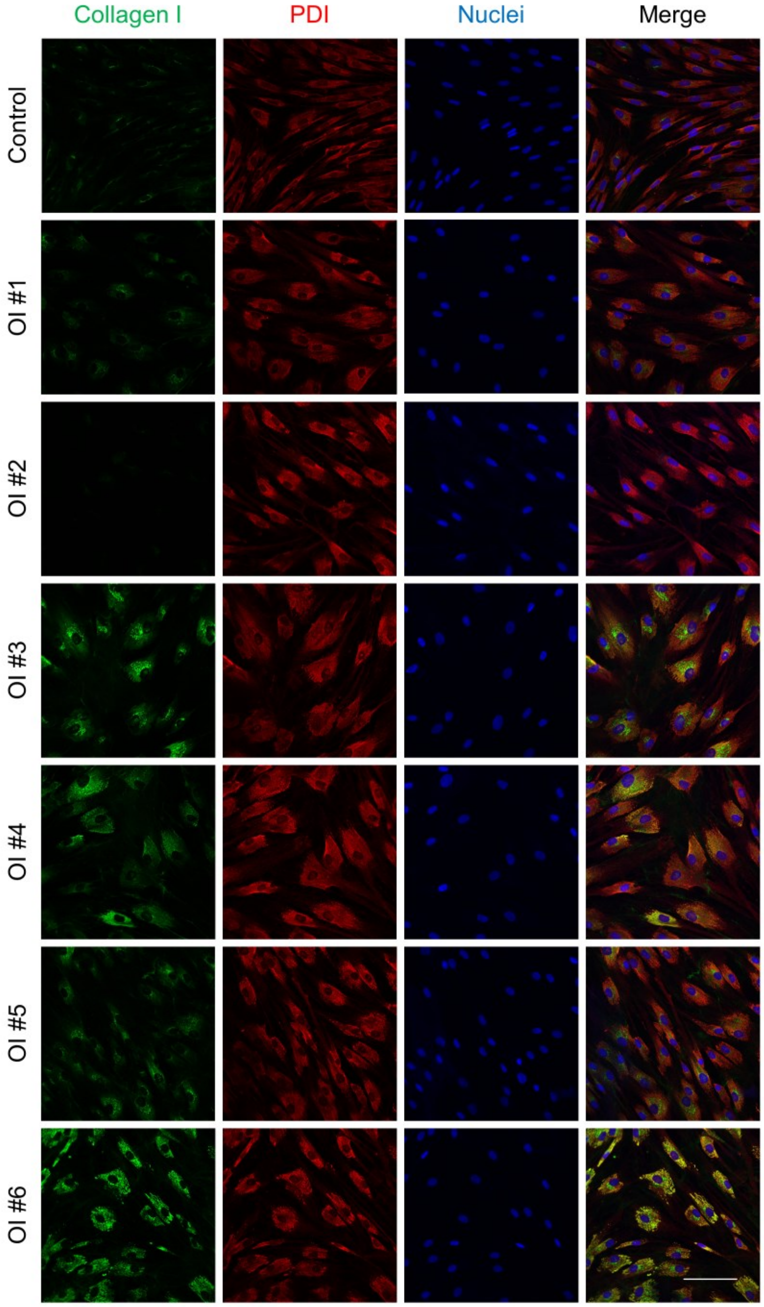

Figure S1. Immunofluorescence staining of dermal fibroblasts. Type I collagen is in green. Endoplasmic reticulum marker protein, disulfide isomerase (PDI), is in red. Nuclei are in blue. Image panels of Control, OI #2 and OI #3 are redisplays of image panels of Figure 1. Scale bar is 100 µm.

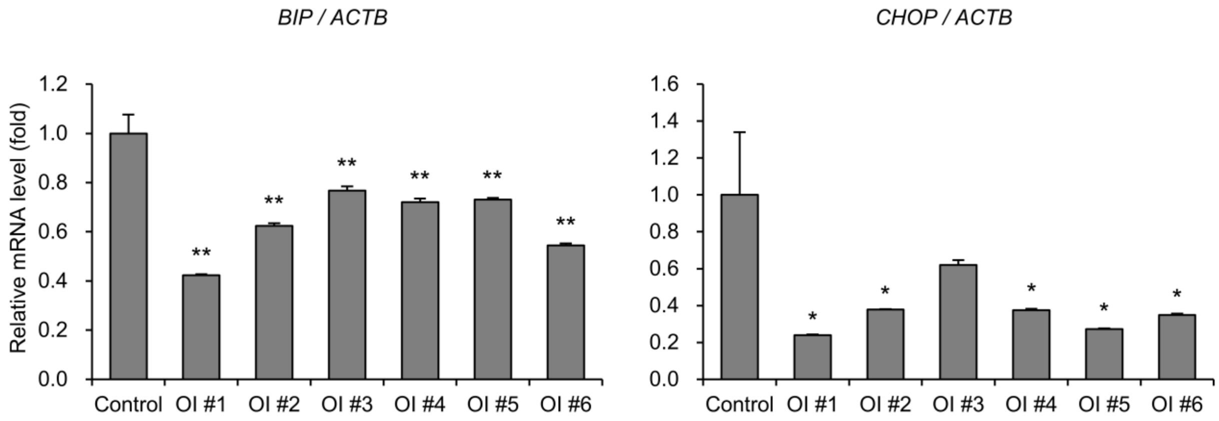

Figure S2. Real-time quantitative PCR of *BIP* and *CHOP*. Data are mean ± SEM. Differences were tested by ANOVA followed by Tukey's HSD post hoc test. *$P < 0.05$ vs. control, **$P < 0.01$ vs. control.

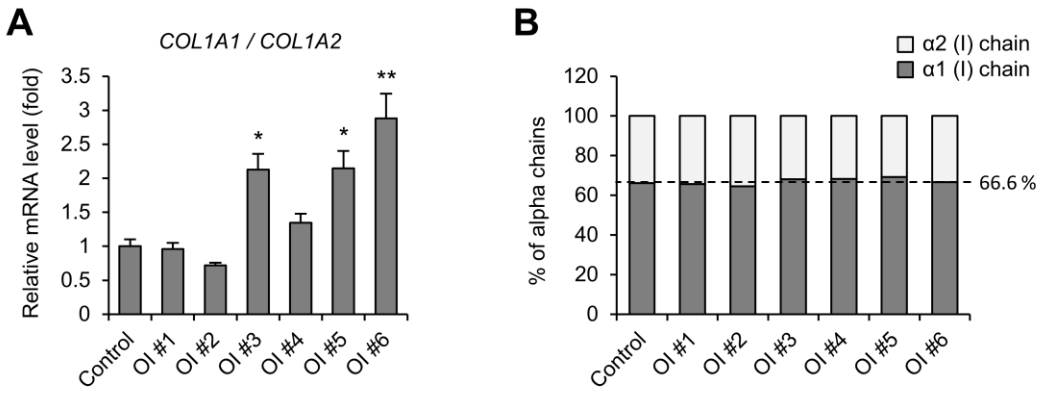

Figure S3. Comparison of α1 and 2 chains of type I collagen. **A.** The ratio of the gene expression of *COL1A1* to *COL1A2*. **B.** The percentage of α1 and 2 chains of type I collagen in conditioned medium. Data are mean ± SEM. Differences were tested by ANOVA followed by Tukey's HSD post hoc test. *$P < 0.05$ vs. control, **$P < 0.01$ vs. control.

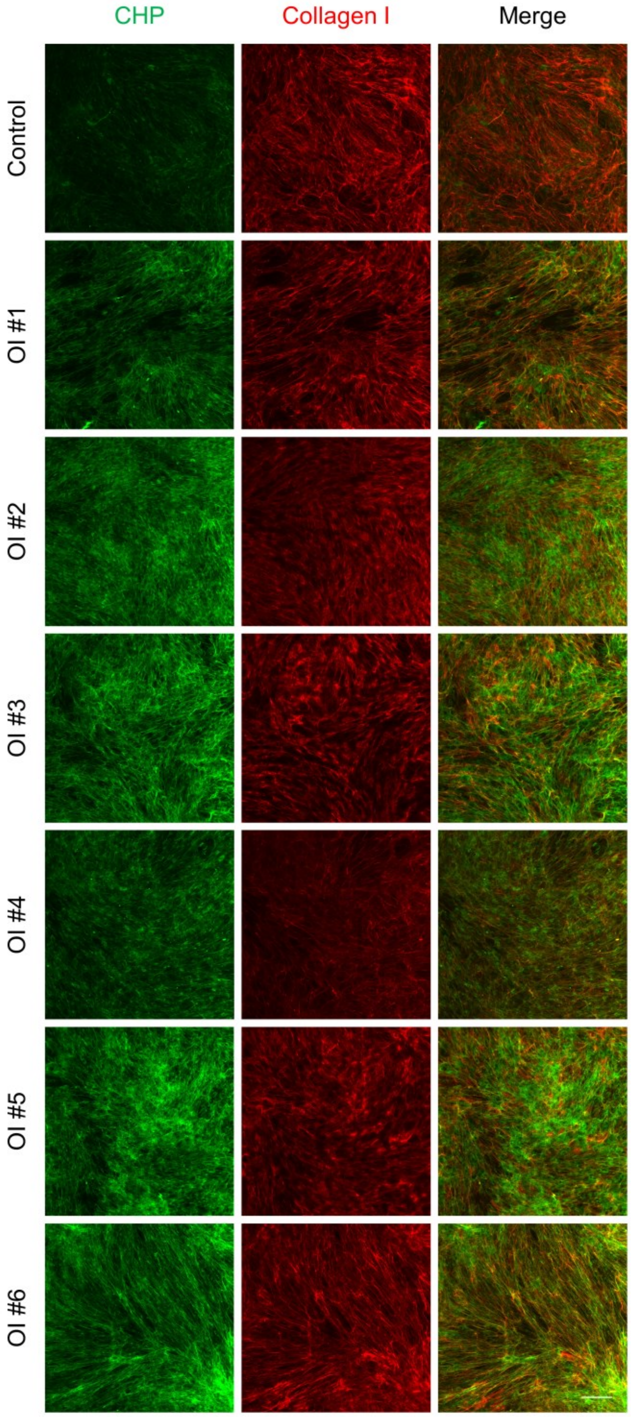

Figure S4. Fluorescence staining of deposited collagen. Unfolded triple-helical chains are stained in green by collagen hybridizing peptide (CHP). Type I collagen is stained in red. Image panels of Control and OI #3 are redisplays of image panels of Figure 4. Scale bar is 200 μm.

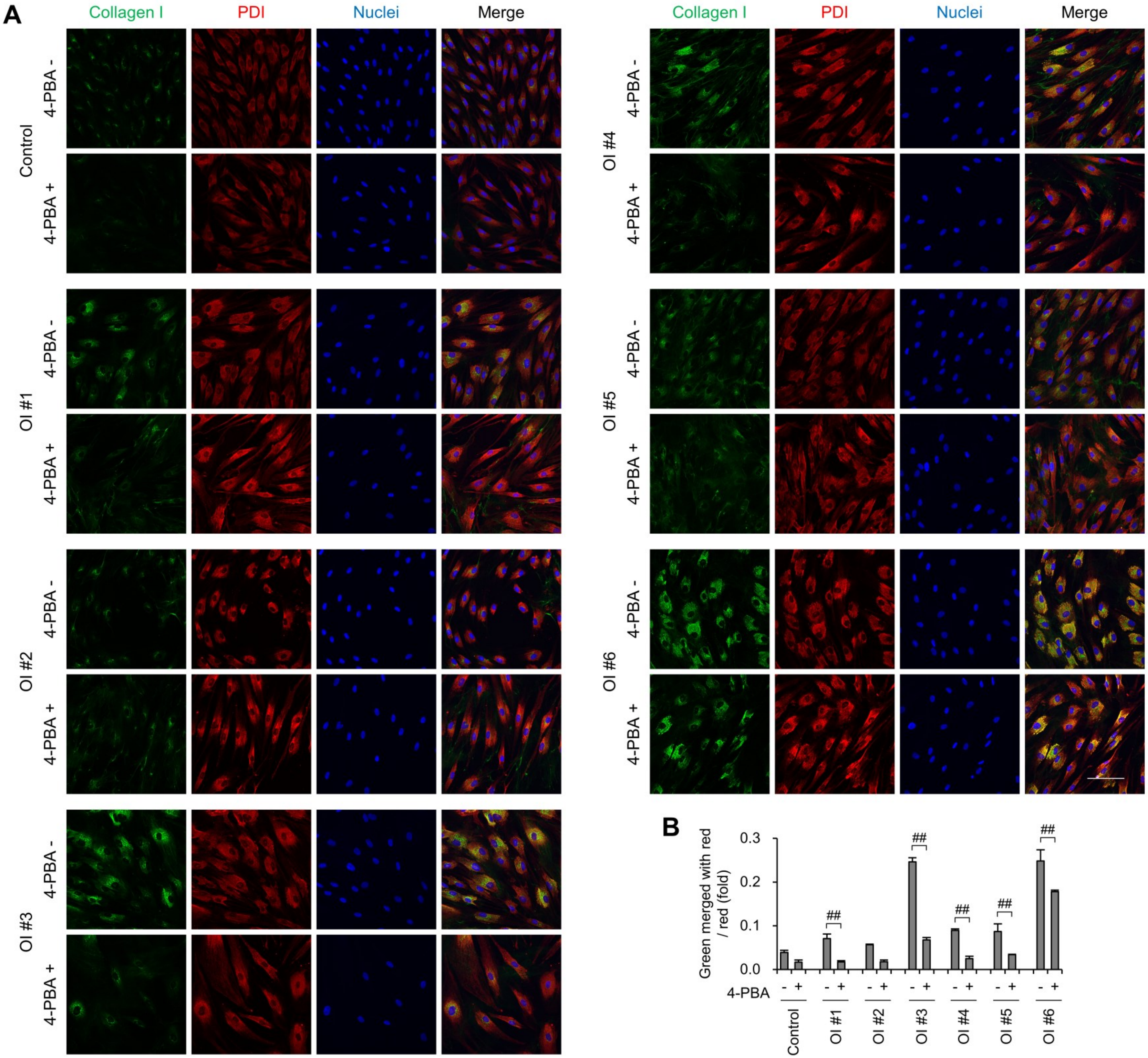

Figure S5. Immunofluorescence staining of dermal fibroblasts treated with or without 5 mM 4-phenylbutyric acid (4-PBA). **A.** Immunofluorescence staining of dermal fibroblasts. Type I collagen is in green. The endoplasmic reticulum marker protein, disulfide isomerase (PDI), is in red. Nuclei are in blue. Image panels of OI #3 are redisplays of image panels of Figure 5. Scale bar is 100 µm. **B.** Calculated values of green area merged with red area divided by red area of immunofluorescence staining. Data are mean ± SEM. Differences were tested by ANOVA followed by Tukey's HSD post hoc test. ##$P < 0.01$.

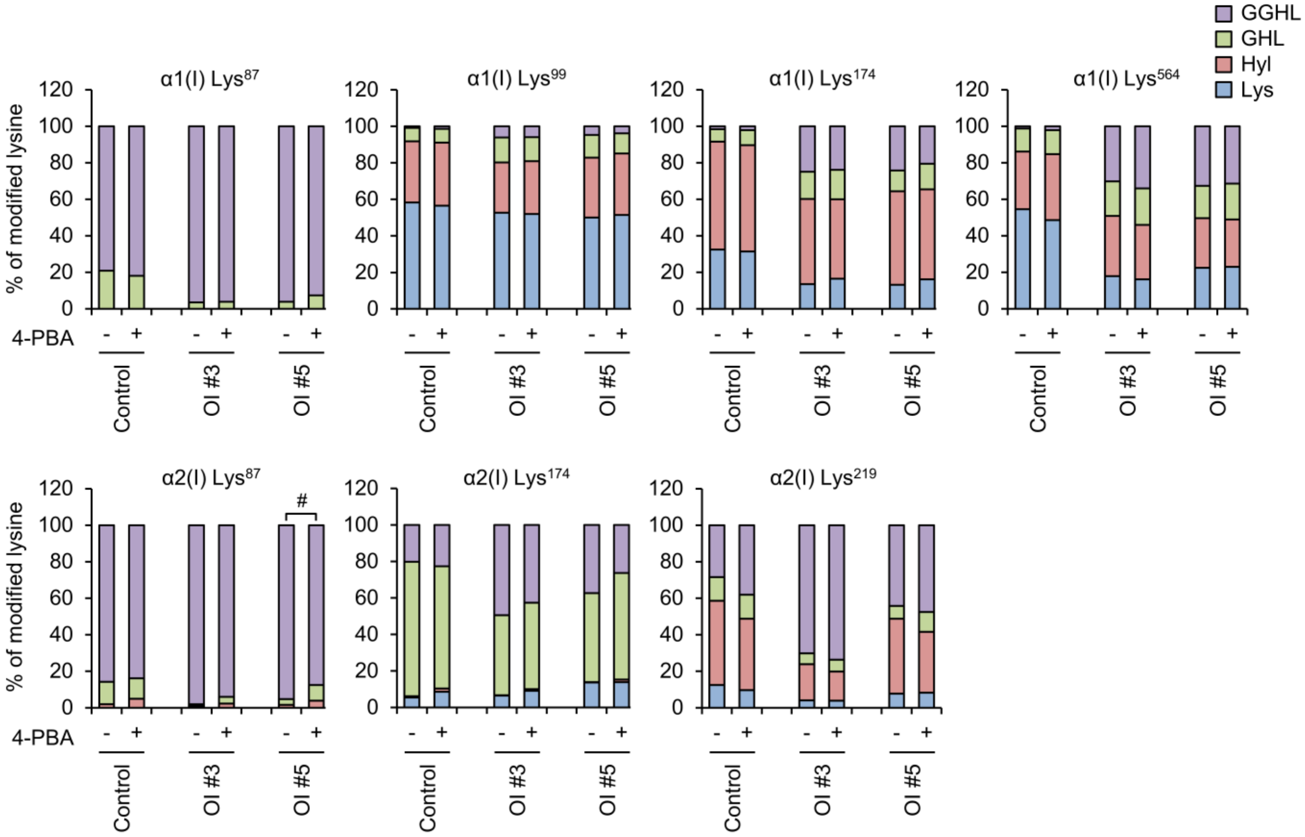

Figure S6. The effect of 4-phenylbutyric acid (4-PBA) on post-translational modification of type I collagen. Each cell line was treated with or without 5 mM 4-PBA. The percentage of post-translational modifications at Lys^{87}, Lys^{99}, Lys^{174}, and Lys^{564} of α1 and Lys^{87}, Lys^{174}, and Lys^{219} of α2 chain of type I collagen analyzed by LC-MS. Control is the average of 2 cell lines. Hyl, hydroxylysine; GHL, galactosyl-hydroxylysine; GGHL, glucosyl-galactosyl-hydroxylysine. Differences in the ratio of GGHL to non-GGHL were tested by Pearson's chi-square test. #$P < 0.05$, ##$P < 0.01$.

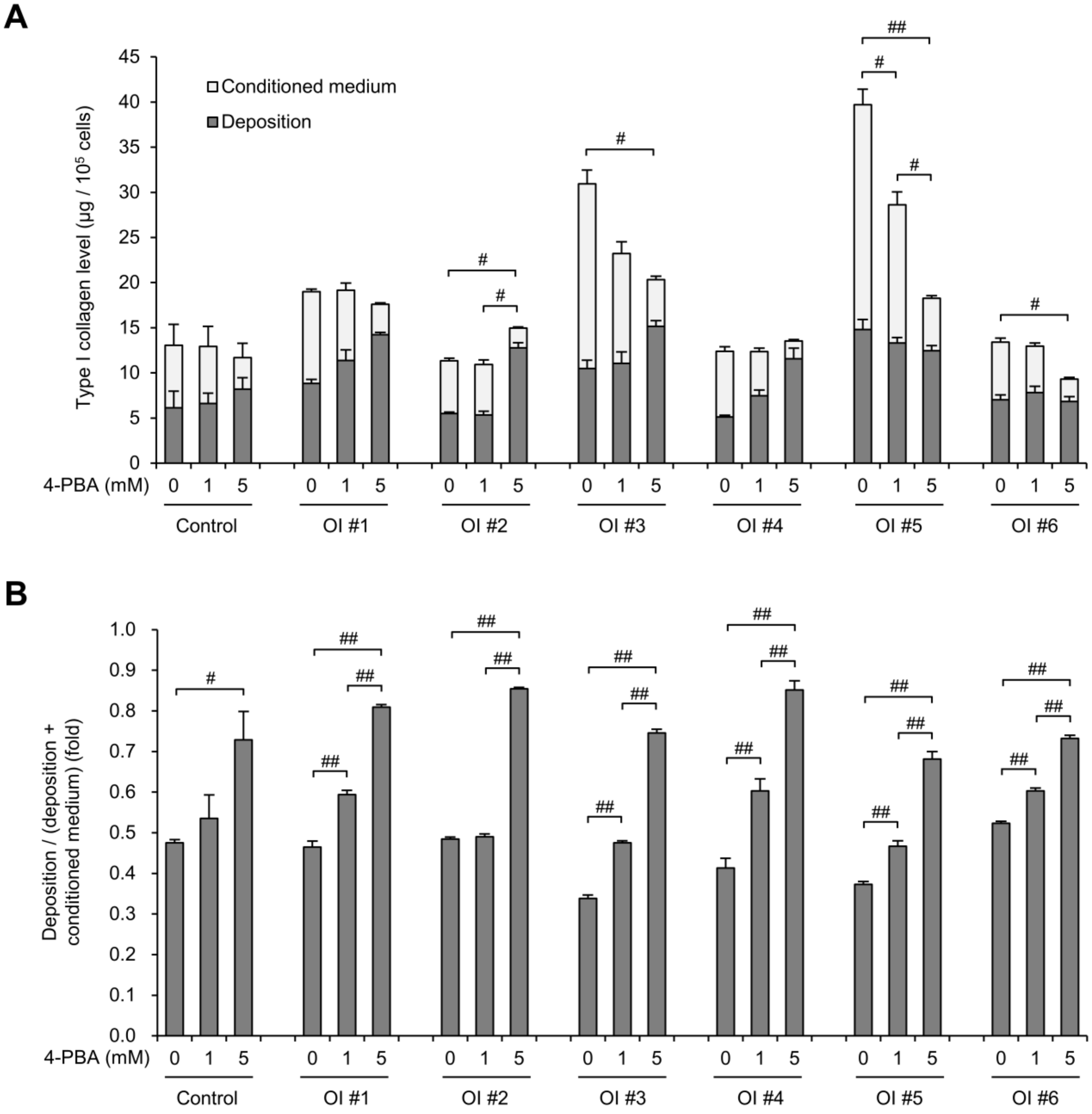

Figure S7. The effect of 4-phenylbutyric acid (4-PBA) on the production of type I collagen. **A.** Protein levels of type I collagen in conditioned medium and deposited on the dish measured by ELISA. **B.** Calculated values of protein levels of type I collagen. The formula for calculation is as follows: protein levels deposited on the dish/(protein levels deposited on the dish + protein levels in conditioned medium). All data are mean ± SEM. Differences were tested by ANOVA followed by Tukey's HSD post hoc test. #*P* < 0.05, ##*P* < 0.01.

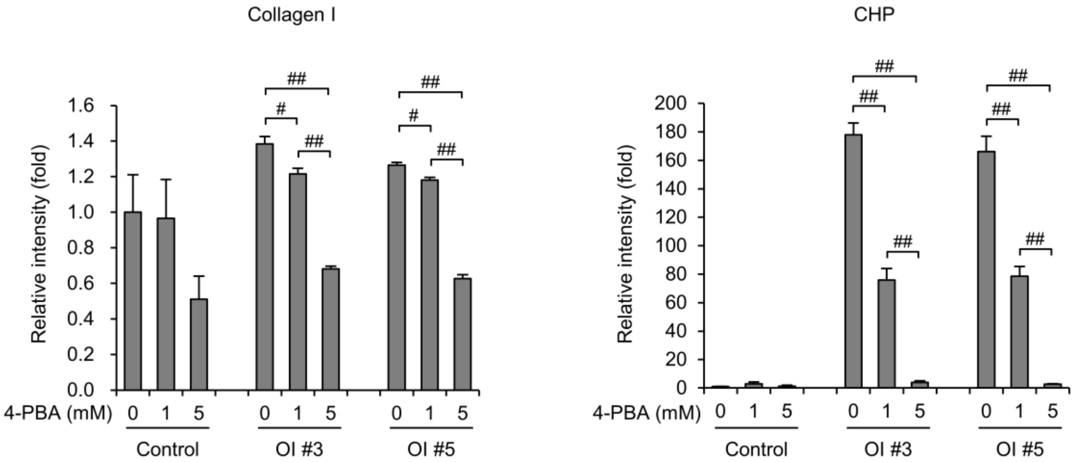

Figure S8. Calculated values of fluorescence staining. The formula for calculation is as follows: red area × red fluorescence density for type I collagen and green area × green fluorescence density for CHP. Data are mean ± SEM. Differences were tested by ANOVA followed by Tukey's HSD post hoc test. #$P < 0.05$, ##$P < 0.01$.

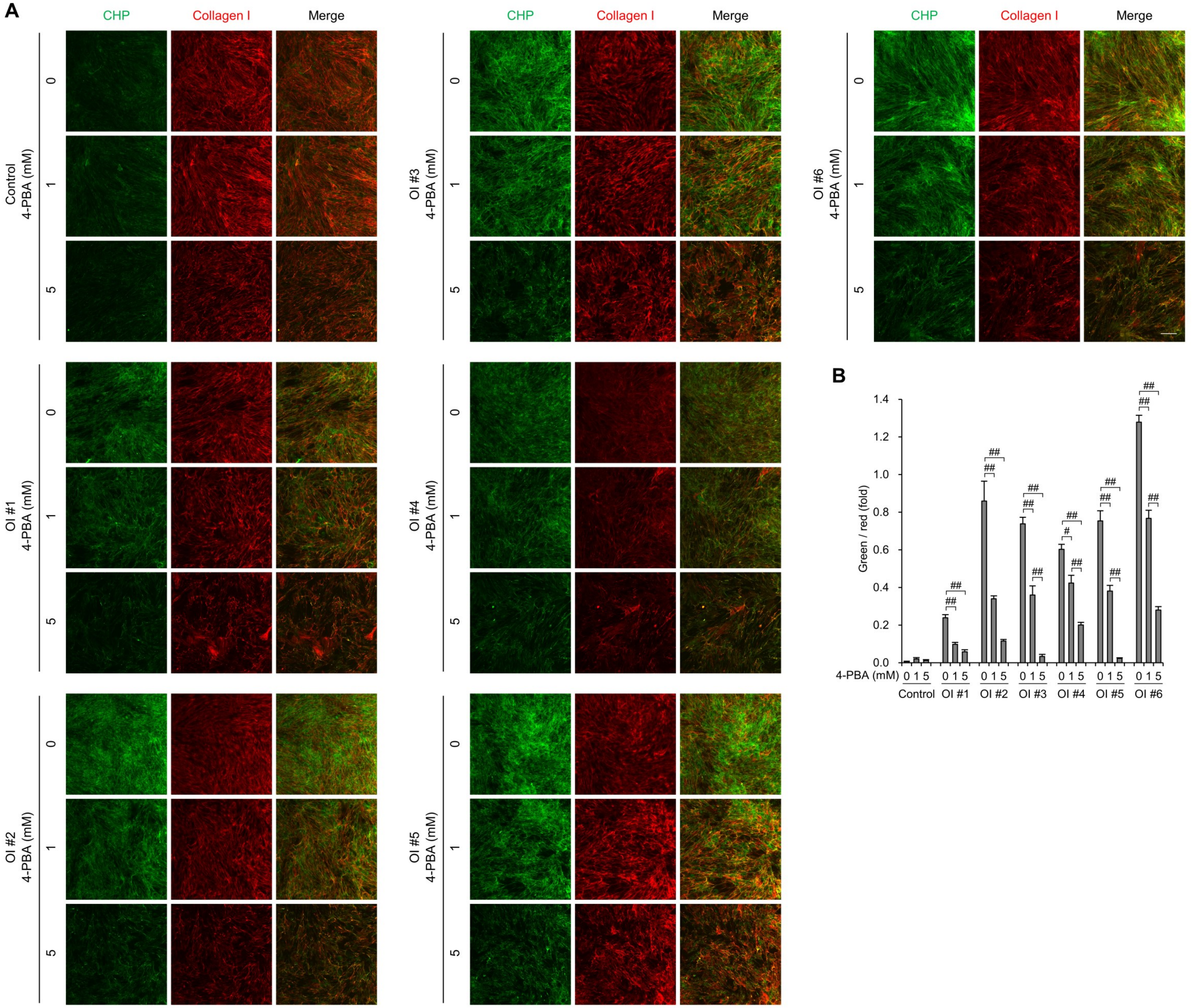

Figure S9. The effect of 4-phenylbutyric acid (4-PBA) on type I collagen in the extracellular matrix. **A.** Fluorescence staining of deposited collagen. Misfolded triple-helical chains are stained in green by collagen hybridizing peptide (CHP). Type I collagen is stained red. Image panels of OI #3 are redisplays of image panels of Figure 7. Scale bar is 200 µm. **B.** Calculated values of fluorescence staining. The formula for calculation is as follows: (green area × green fluorescence density)/(red area × red fluorescence density). Data are mean ± SEM. Differences were tested by ANOVA followed by Tukey's HSD post hoc test. #$P < 0.05$, ##$P < 0.01$.

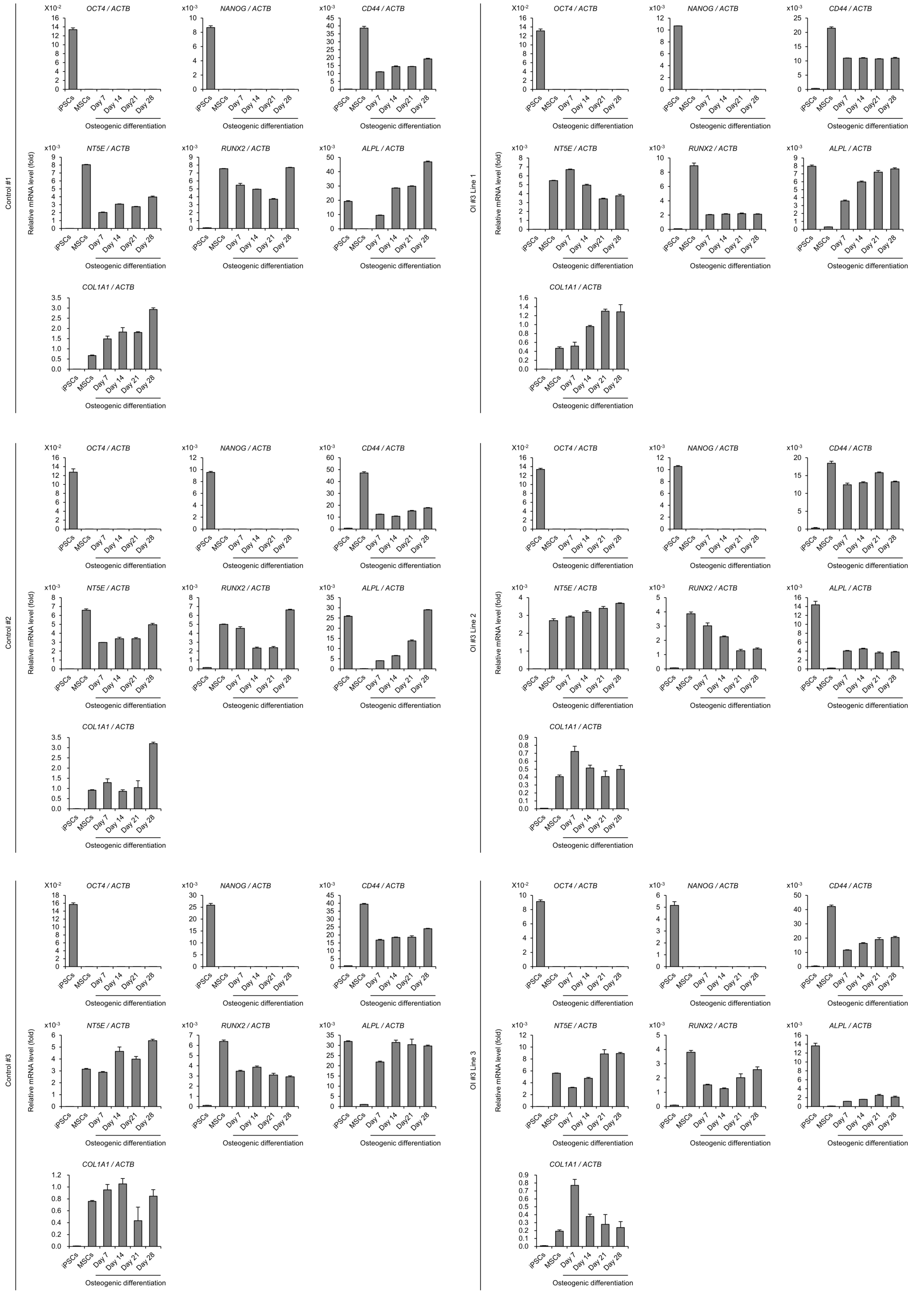

Figure S10. Gene expression of control and OI #3 cell lines. Pluripotency markers, *OCT4* and *NANOG,* mesenchymal stromal cell (MSC) markers, *CD44* and *NT5E,* and osteogenic markers, *RUNX2, ALPL,* and *COL1A1* were checked in induced pluripotent stem cells (iPSCs) and MSCs and after osteogenic differentiation for 7, 14, 21, and 28 d by real-time quantitative PCR. The values are indicated as relative gene expression using *ACTB* as a reference gene. All data are mean ± SEM.

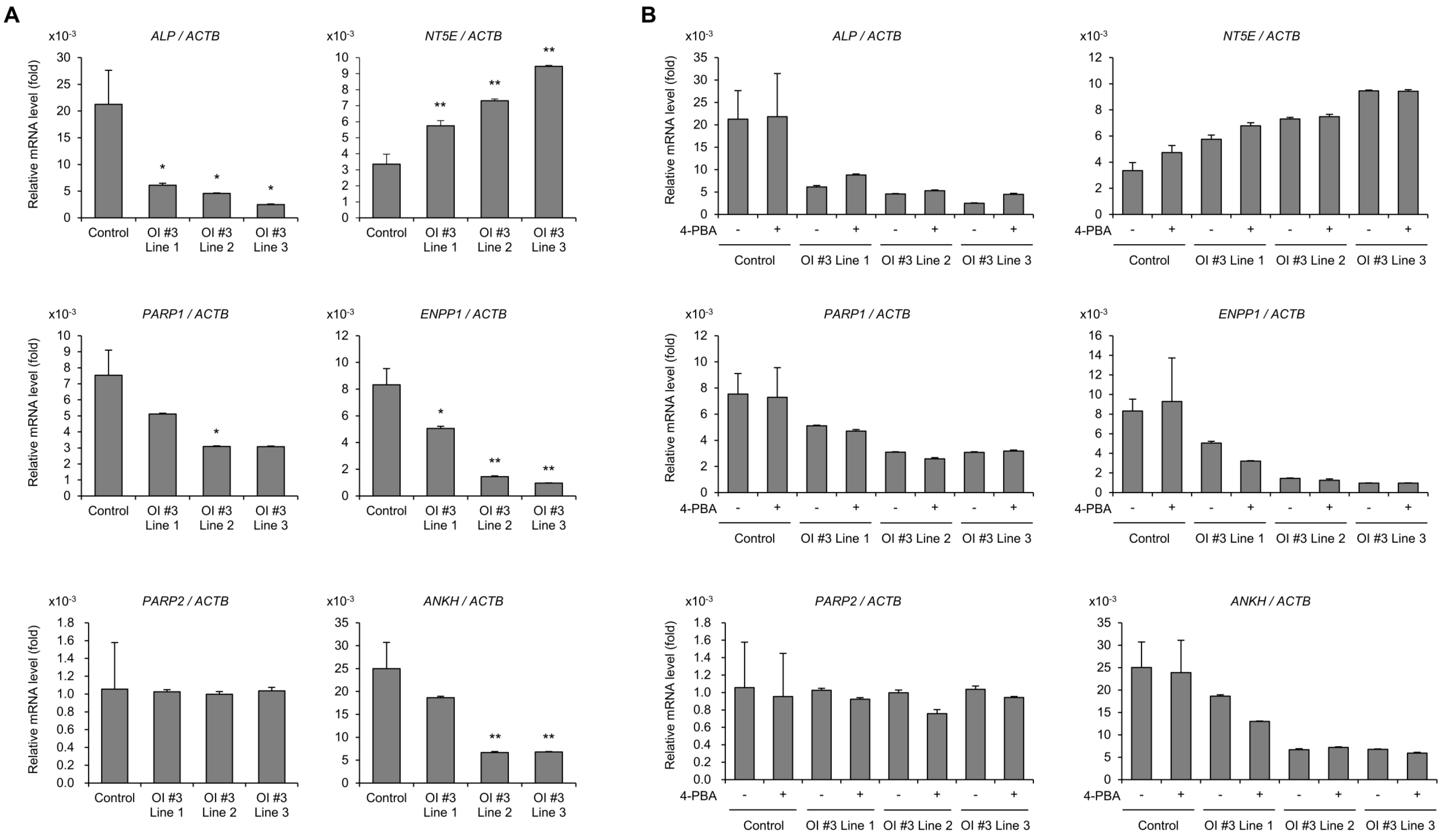

Figure S11. Gene expression of control and OI #3 cell lines. Pro-calcification genes, *ALPL, PARP1,* and *PARP2,* and anti-calcification genes, *NT5E, ENPP1,* and *ANKH,* were checked at day 14 following osteoblast differentiation with no 4-phenylbutyric acid (4-PBA) **(A)** and with or without 4-PBA **(B)** by real-time quantitative PCR. The values are indicated as relative gene expression using *ACTB* as a reference gene. All data are mean ± SEM. Differences were tested by ANOVA followed by Tukey's HSD post hoc test. *$P < 0.05$ vs. control, **$P < 0.01$ vs. control.

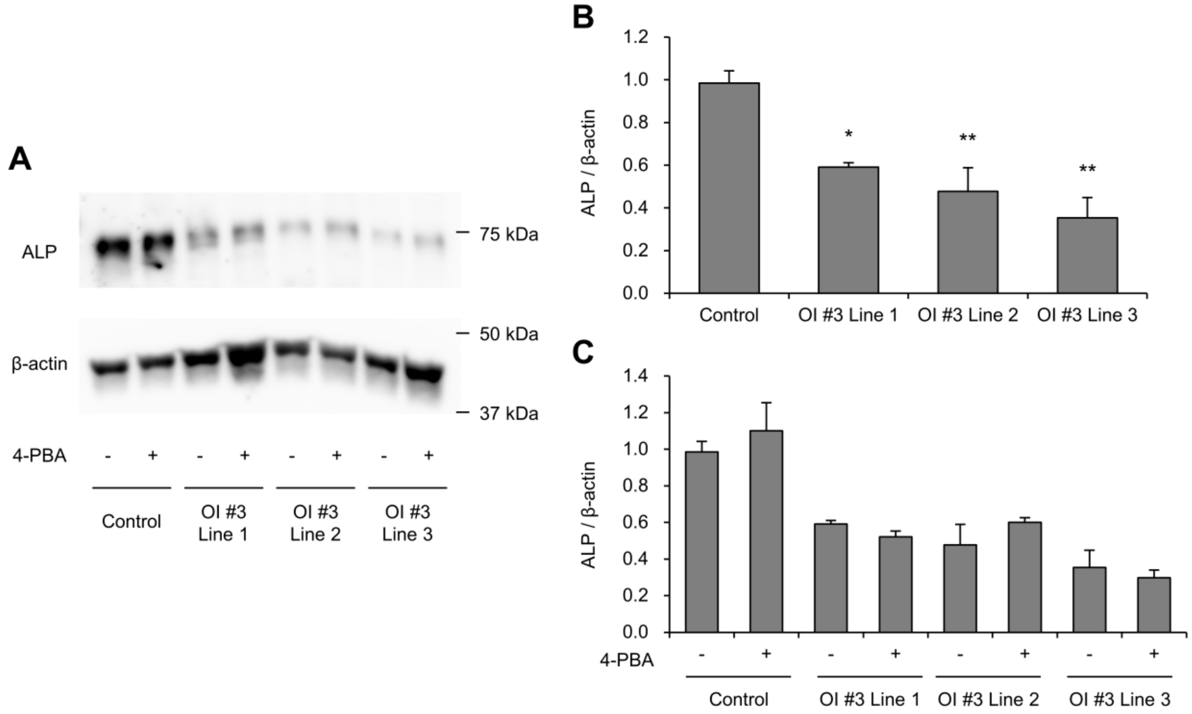

Figure S12. Protein level of alkaline phosphatase (ALP) in control and OI #3 cell lines. **A.** Western blotting of alkaline phosphatase and β-actin at day 14 following osteoblast differentiation with or without 4-phenylbutyric acid (4-PBA). **B and C.** ALP levels are indicated as the relative levels of β-actin quantified by densitometry. All data are mean ± SEM. Differences were tested by ANOVA followed by Tukey's HSD post hoc test. *$P < 0.05$ vs. control, **$P < 0.01$ vs. control.

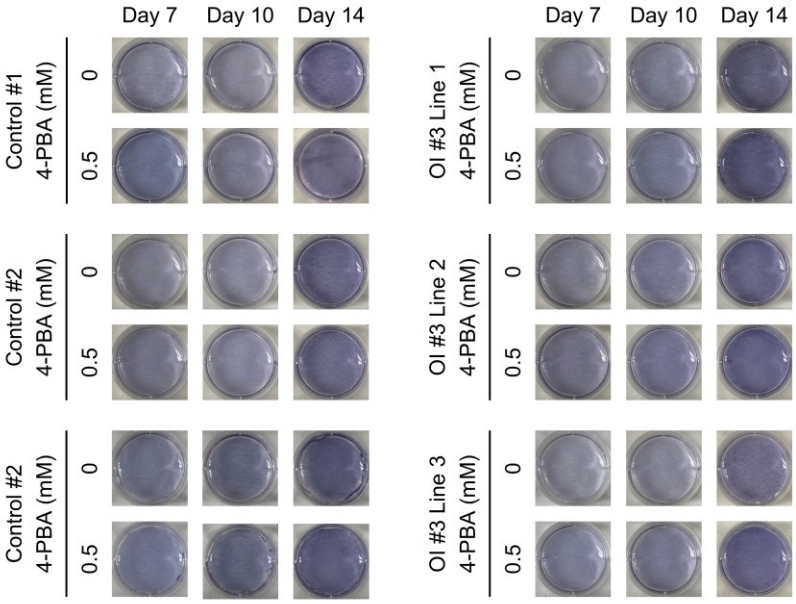

Figure S13. Alkaline phosphatase staining in induced osteoblasts. Induction of osteoblast differentiation from mesenchymal stromal cells was performed during the indicated day with or without 4-phenylbutyric acid (4-PBA).

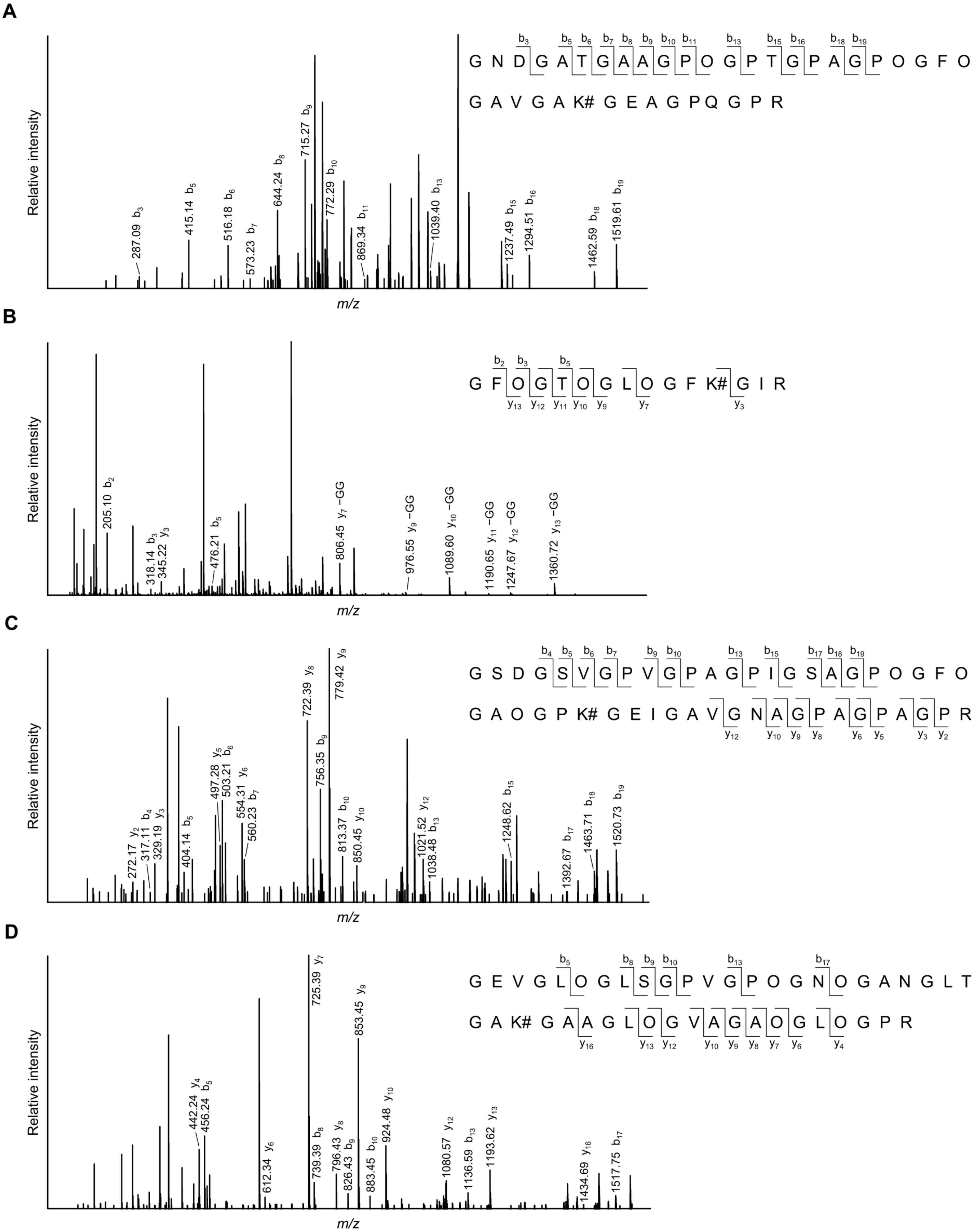

Figure S14. MS/MS spectra of GGHL-containing tryptic peptides in type I collagen. **A.** α1(I) [145–183] GNDGATGAAGPOGPTGPAGPOGFOGAVGAK#^{174}GEAGPQGPR (z = 4, m/z 935.1465; O indicates Hyp, and K# indicates GGHL). **B.** α2(I) [76–90] GFOGTOGLOGFK#^{87}GIR (z = 3, m/z 630.3103). **C.** α2(I) [145–192] GSDGSVGPVGPAGPIGSAGPOGFOGAOGPK#^{174}GEIGAVGNAGPAGPAGPR (z = 4, m/z 1115.5401). **D.** α2(I) [193–237] GEVGLOGLSGPVGPOGNOGANGLTGAK#^{219}GAAGLOGVAGAOGLOGPR (z = 4, m/z 1076.2862). −GG represents deglycosylated fragment ions.
